# Supplementary material for: Temperature sensitive liposome based cancer nanomedicine enables tumour lymph node immune microenvironment remodelling
Source: Nat Commun. 2023 Apr 19;14:2248. doi: 10.1038/s41467-023-38014-6 (PMC10115817; doi:10.1038/s41467-023-38014-6)
Supplement: Supplementary file 1 — Supplementary Information [file 41467_2023_38014_MOESM1_ESM.pdf]

Temperature Sensitive Liposome Based Cancer Nanomedicine Enables Tumour Lymph Node Immune Microenvironment Remodelling

Shunli Fu[1], Lili Chang[1], Shujun Liu[1], Tong Gao[1], Xiao Sang[1], Zipeng Zhang[1], Weiwei Mu[1], Xiaoqing Liu[1], Shuang Liang[1], Han Yang[1], Huizhen Yang[1], Qingping Ma[1], Yongjun Liu [1,*], Na Zhang[1,*]

*Corresponding author.

E-mail: liuyongjun@sdu.edu.cn (Yongjun Liu); zhangnancy9@sdu.edu.cn (Na Zhang)

[1]NMPA Key Laboratory for Technology Research and Evaluation of Drug Products and Key Laboratory of Chemical Biology (Ministry of Education), Department of Pharmaceutics, School of Pharmaceutical Sciences, Cheeloo College of Medicine, Shandong University, 44 Wenhuaxi Road, Jinan, Shandong Province 250012, China

Table of contents

Figure S1. Characterization of DSPE-PEG_{2000}-MMP2-sensitive pep-Mal and DSPE-PEG_{2000}-MMP2-non-sensitive pep-Mal.

Figure S2. Characterization of DSPE-PEG_{2000}-MMP2-sensitive pep-BSA.

Figure S3. Characterization of DSPE-Hyd-PEG_{5000}-NGR.

Figure S4. N-I-Lip-S showed similar tumour accumulation and more LNs accumulation than N-I-Lip-L.

Figure S5. N-I-Lip with 1/8 mass ratio of cholesterol showed more LNs accumulation.

Figure S6. Migration of N-I-Lip-S from tumour to the inguinal LNs.

Figure S7. N-I-Lip with 1/8 mass ratio of cholesterol showed better tumour accumulation and more LNs accumulation both in the lipid material of lecithin high potency and egg yolk lecithin.

Figure S8. Evaluation of IDO1 expression both in LNs and tumour after photothermal therapy.

Figure S9. The UV-Vis absorption spectra of free 1-MT, free IR780 and NIL-IM-Lip.

Figure S10. The TEM morphology of NIL-IM-Lip after irradiation of 808 nm laser (1.0 W/cm^2, 5 min).

Figure S11. The HPLC chromatograms of MMP2 enzyme alone and The HPLC chromatograms of 1.0 mg/mL MMP2 sensitive peptide after incubated with different concentrations MMP2 enzyme.

Figure S12. The free IR780 showed good photothermal stability.

Figure S13. N-I-Lip and NIL-I-Lip showed similar tumour and LNs accumulation.

Figure S14. The Infrared thermal imaging and the temperature profile of tumour under 0.6 and 1.0 W/cm^2 laser.

Figure S15. NIL-IM-lip induced stronger ROS generation in *vitro*.

Figure S16. The purity of NK cells separated by MACS method.

Figure S17. Tumour volume curves of different formulation-treated B16F10 tumour-bearing C57BL/6 mice (n=6).

Figure S18. The individual tumour growth curves of B16F10-bearing C57BL/6 mice after intravenous injection with different formulations.

Figure S19. NIL-IM-Lip treatment with laser irradiation showed enhanced antitumour efficacy compared with NIL-IM-Lip without laser irradiation.

Figure S20. Quantification of (a) Ki67 and (b) TUNEL staining assays of tumour tissues.

Figure S21. The evaluation of hemolysis assay of NIL-IM-Lip *in vitro.*

Figure S22. The H&E staining of heart, liver, spleen, lung and kidneys from B16F10 bearing mice with different formulations treatment.

Figure S23. The establishment process of postsurgical model.

Figure S24. The individual tumour growth curves of B16F10-bearing C57BL/6 mice in postsurgical model.

Figure S25. NIL-IM-Lip with laser showed enhanced antitumour efficacy than NIL-IM-Lip without laser in postsurgical model.

Figure S26. The ratio of Kyn and Trp in tumour tissues obtained from B16F10-bearing mice were determined by HPLC method.

Figure S27. The level of cytokines in tumour tissues.

Figure S28. Evaluation of CRT exposure in tumour tissues.

Figure S29. Evaluation of HMGB1 release in tumour tissues.

Figure S30. Flow cytometric apoptosis analysis of CT26 cells after treatment with PBS, IR780, IR780+L, IR780+1-MT+L, IR780+1-MT+IL-15+L and NIL-IM-Lip+L.

Figure S31. Flow cytometric apoptosis analysis of MC38 cells after treatment with PBS, IR780, IR780+L, IR780+1-MT+L, IR780+1-MT+IL-15+L and NIL-IM-Lip+L.

Figure S32. Fluorescence microscopy images of CRT exposure to CT26 cells.

Figure S33. Fluorescence microscopy images of CRT exposure to MC38 cells.

Figure S34. The percent survival of NS group, NIL-IM-Lip+L group, NIL-IM-Lip+PD-1+L group on both B16F10 model and CT26 model.

Figure S35. Gating strategies for immune cells in LNs.

Figure S36. Gating strategies for immune cells in tumours.

Figure S37. Gating strategies for memory T cells in spleen ($CD3^{+}CD8^{+}CD44^{+}CD62L^{-}$).

Table S1. The characteristic of NIL-IM-Lip with different particle size and mass ratio.

Table S2. The characteristic of NIL-IM-Lip

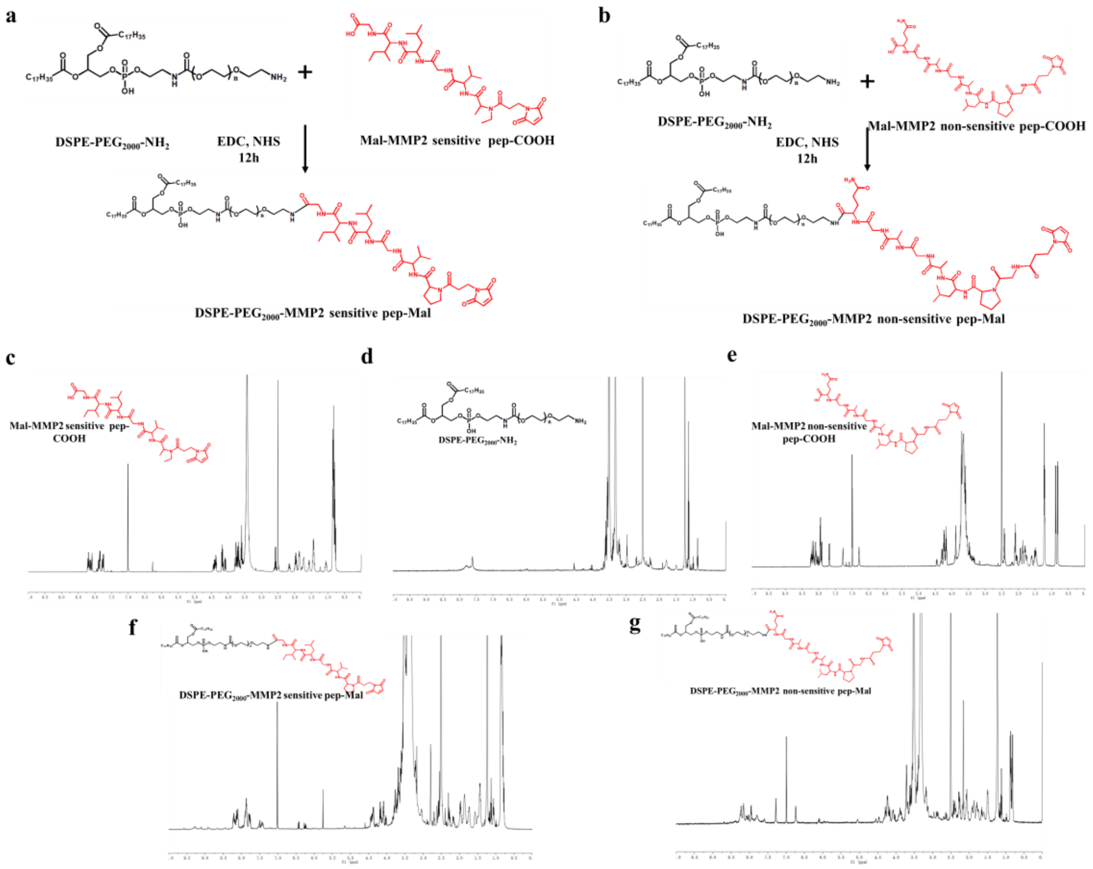

Supplementary Fig. 1. Characterization of DSPE-PEG$_{2000}$-MMP2-sensitive pep-Mal and DSPE-PEG$_{2000}$-MMP2-non-sensitive pep-Mal. a, Synthetic route to prepare DSPE-PEG$_{2000}$-MMP2-sensitive pep-Mal. **b,** Synthetic route to prepare DSPE-PEG$_{2000}$-MMP2 non-sensitive pep-Mal. **c,** ^{1}H NMR spectrum of Mal-MMP2-sensitive pep-COOH. **d,** ^{1}H NMR spectrum of DSPE-PEG$_{2000}$-NH_2. **e,** ^{1}H NMR spectrum of Mal-MMP2 non-sensitive pep-COOH. **f,** The ^{1}H NMR spectrum of DSPE-PEG$_{2000}$-MMP2-sensitive pep-Mal showed the peak from Mal at 7.0 ppm and the peak from PEG from 3.25 ppm to 3.75 ppm, indicating the successful synthesis of DSPE-PEG$_{2000}$-MMP2-sensitive pep-Mal. **g,** The ^{1}H NMR spectrum of DSPE-PEG$_{2000}$-MMP2 non-sensitive pep-Mal showed the characteristic peak of Mal-MMP2 non-sensitive pep-COOH from 6.5 ppm to 7.5 ppm and the peak from PEG from 3.25 ppm to 3.75 ppm, indicating the successful synthesis of DSPE-PEG$_{2000}$-MMP2 non-sensitive pep-Mal. ^{1}H NMR: ^{1}H nuclear magnetic resonance.

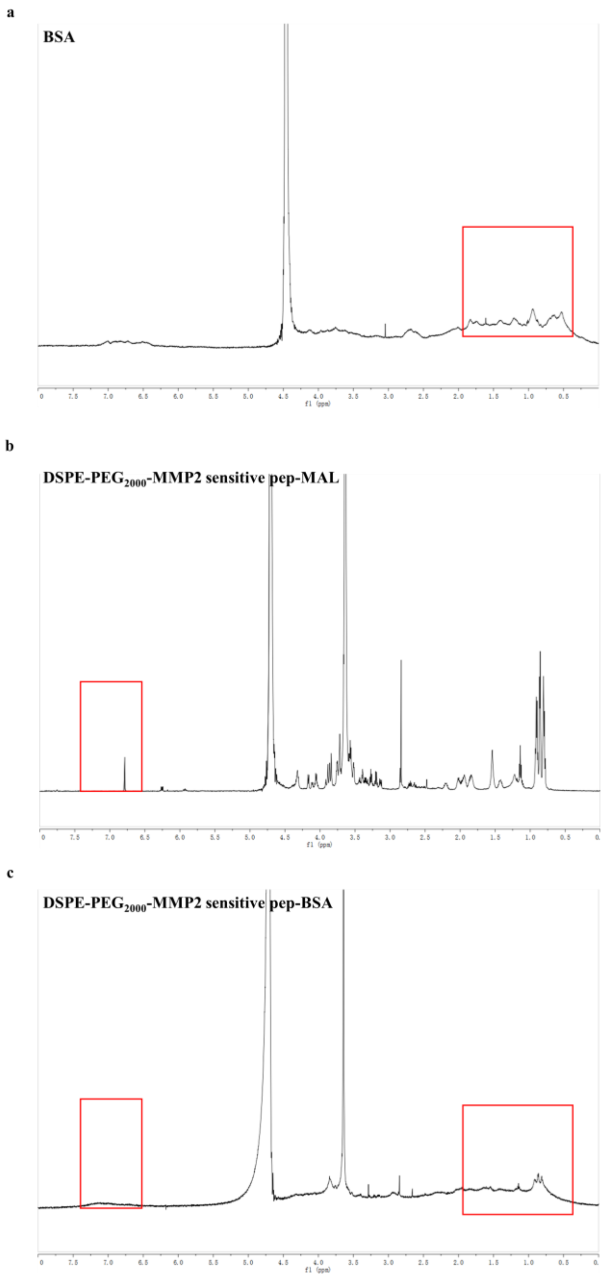

Supplementary Fig. 2. Characterization of DSPE-PEG$_{2000}$-MMP2-sensitive pep-BSA. a, ^{1}H NMR spectrum of BSA. **b,** ^{1}H NMR spectrum of DSPE-PEG$_{2000}$-MMP2-sensitive pep-Mal. **c,** ^{1}H NMR spectrum of DSPE-PEG$_{2000}$-MMP2 non-sensitive pep-BSA showed that the peak from Mal at 7.0 ppm disappeared and the characteristic peak of BSA from 0.5 ppm to 2.0 ppm appeared, indicating the successful synthesis of DSPE-PEG$_{2000}$-MMP2 sensitive pep-BSA.

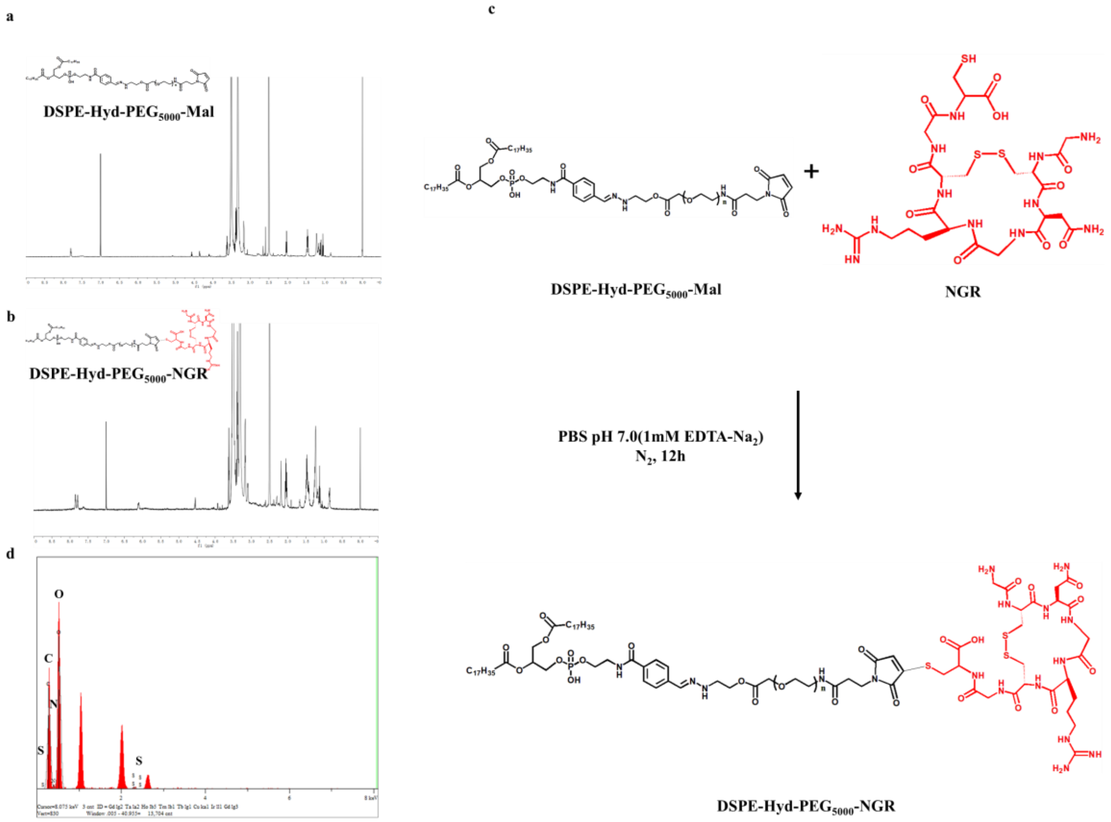

Supplementary Fig. 3. Characterization of DSPE-Hyd-PEG$_{5000}$-NGR. a, ^{1}H NMR spectrum of DSPE-Hyd-PEG$_{5000}$-Mal. **b,** The ^{1}H NMR spectrum of DSPE-Hyd-PEG$_{5000}$-NGR showed that the peak from Mal at 7.0 ppm disappeared and the characteristic peaks of NGR from 1.5 ppm to 1.8 ppm and PEG from 3.25 ppm to 3.75 ppm appeared, indicating the successful synthesis of DSPE-Hyd-PEG$_{5000}$-NGR. **c,** Synthetic route to prepare DSPE-Hyd-PEG$_{5000}$-Mal. **d,** EDS of DSPE-Hyd-PEG$_{5000}$-NGR.

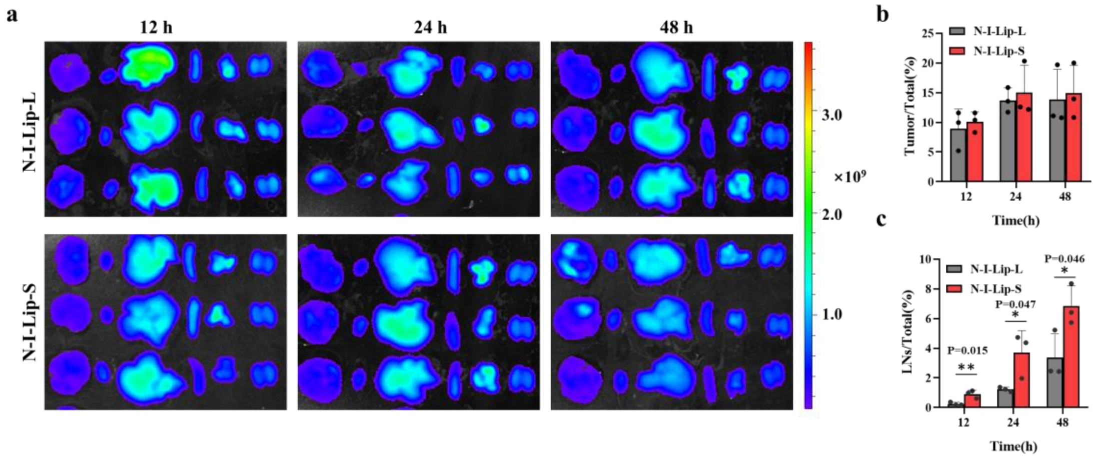

Supplementary Fig. 4. N-I-Lip-S showed similar tumour accumulation and more LNs accumulation than N-I-Lip-L. a, *Ex vivo* imaging of the tumour, heart, liver, spleen, lung and kidneys (from left to right in every picture) at 12, 24, and 48 h post-administration (n = 3 biologically independent animals per group). **b,** Ratio of tumour fluorescence intensity to total fluorescence intensity (n = 3 biologically independent experiments). **c,** Ratio of LNs fluorescence intensity to total fluorescence intensity (n = 3 biologically independent experiments). Data are presented as mean values ± SD. Statistical significance was calculated by the two-tailed Student's t-test. *$P < 0.05$, **$P < 0.01$. Source data are provided as a Source Data file.

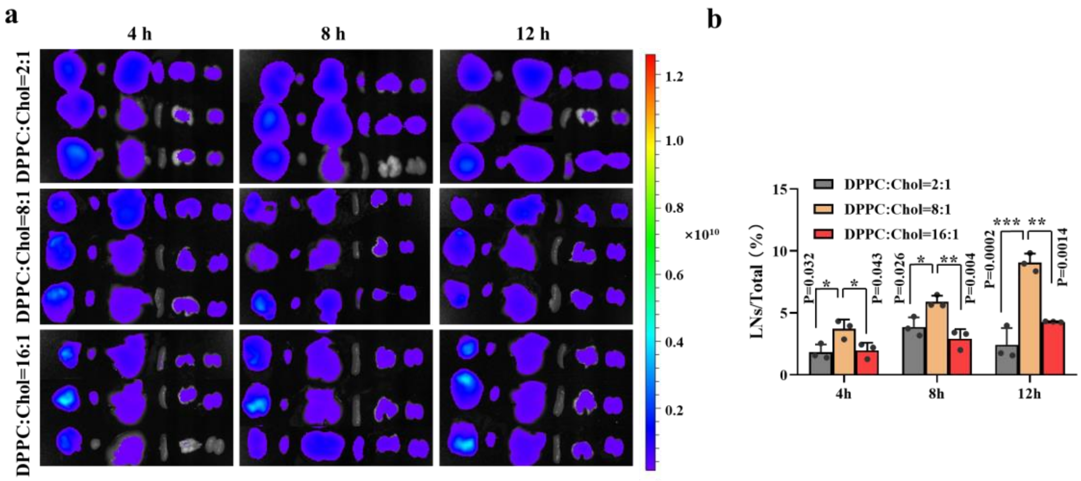

Supplementary Fig. 5. N-I-Lip with 1/8 mass ratio of cholesterol showed more LNs accumulation. a, *Ex vivo* imaging of the tumour, heart, liver, spleen, lung and kidneys (from left to right in every picture) at 4, 8, and 12 h after intratumoural injection (n = 3 biologically independent animals per group). **b,** Ratio of LNs fluorescence intensity to total fluorescence intensity (n = 3 biologically independent experiments). Data are presented as mean values ± SD. Statistical significance was calculated by one-way ANOVA analysis of variance with Tukey's post hoc test. **P* < 0.05, ***P* < 0.01, ****P* < 0.001. Source data are provided as a Source Data file.

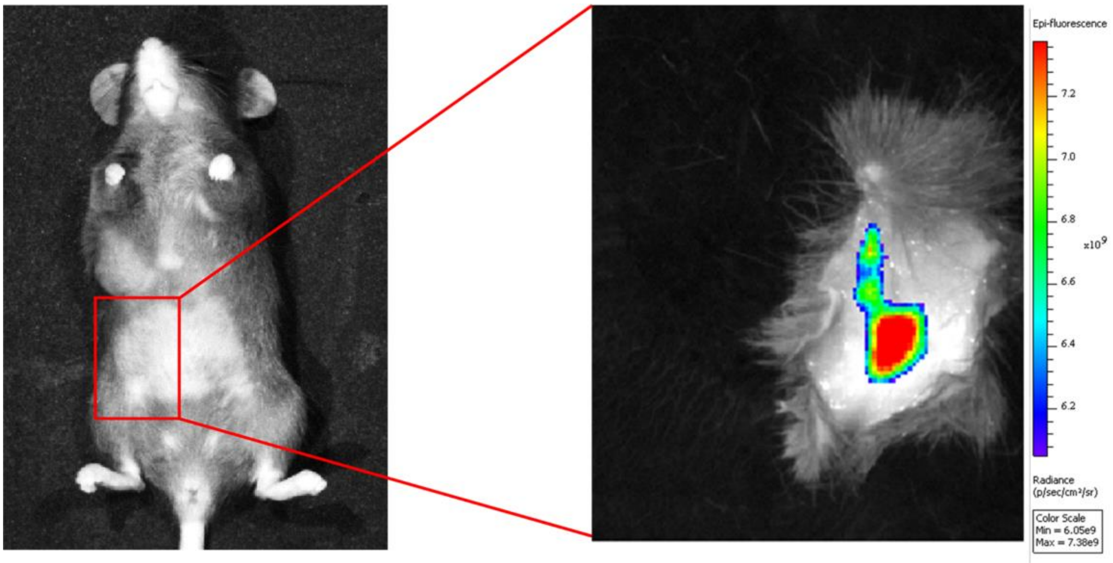

Supplementary Fig. 6. Migration of N-I-Lip-S from the tumour to the inguinal LNs. The mice were sacrificed, and the portion marked in red in the left picture was removed for NIRF imaging. NIRF: near–infrared fluorescence.

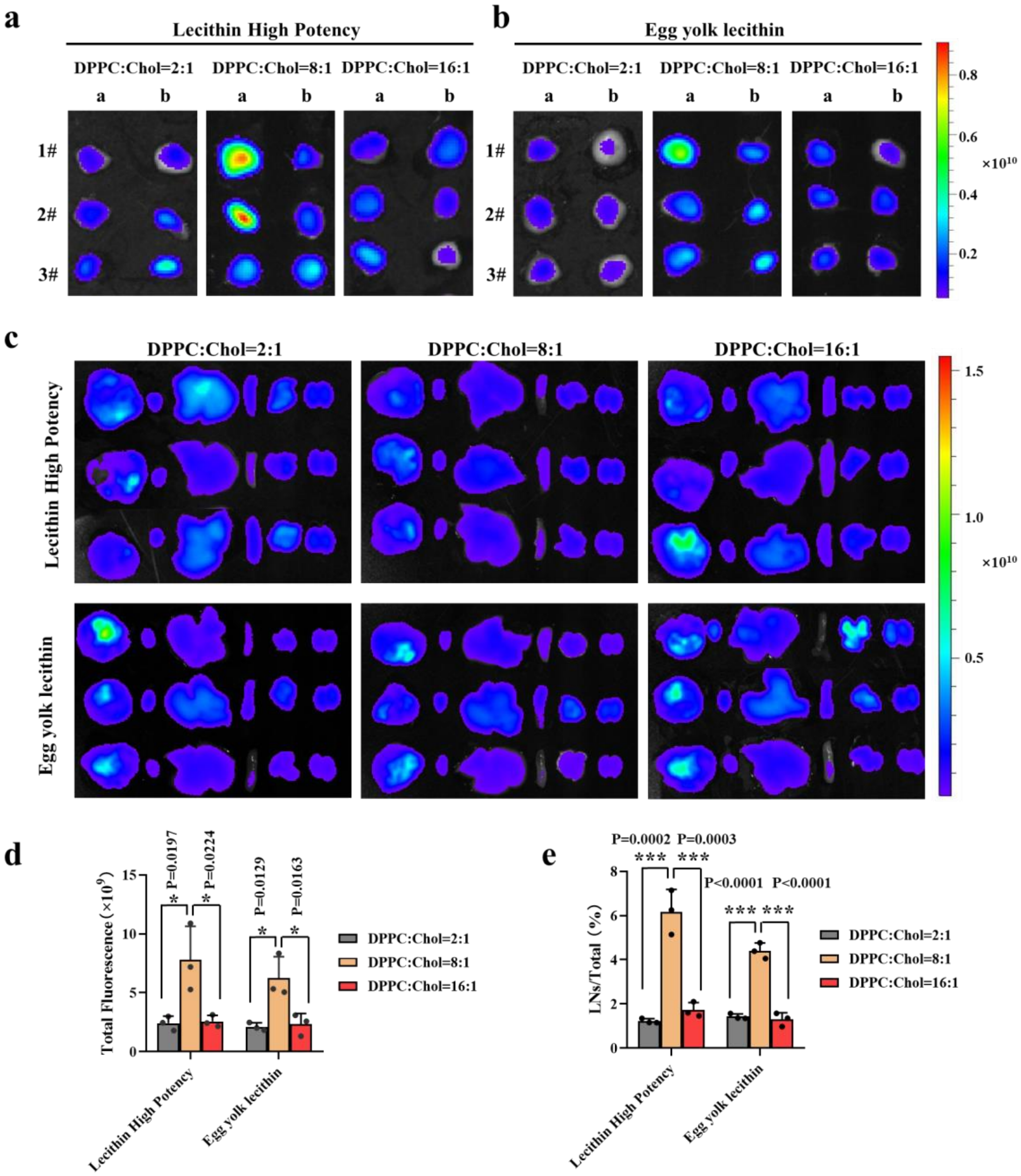

Supplementary Fig. 7. N-I-Lip with 1/8 mass ratio of cholesterol showed better tumour accumulation and more LNs accumulation both in the lipid material of lecithin high potency and egg yolk lecithin. a,b, *Ex vivo* LNs imaging at 12 h post-administration of N-I-Lip with different cholesterol mass ratios both in the lipid material of lecithin high potency (a) and egg yolk lecithin (b) (n = 3 biologically independent animals per group). **c,** *Ex vivo* imaging of the tumour, heart, liver, spleen, lung and kidneys (from left to right in every picture) at 12 h post-administration of N-I-Lip with different cholesterol mass ratios both in the lipid material of lecithin high potency and egg yolk lecithin (n = 3 biologically independent animals per group). **d,** Total fluorescence intensity of the LNs at 12 h post-administration of N-I-Lip with different cholesterol mass ratios both in the lipid material of lecithin high potency and

egg yolk lecithin (n = 3 biologically independent experiments). **e,** Ratio of LNs fluorescence intensity to total fluorescence intensity (n = 3 biologically independent experiments). Data are presented as mean values ± SD. Statistical significance was calculated by one-way ANOVA analysis of variance with Tukey's post hoc test. $*P < 0.05$, $***P < 0.001$. Source data are provided as a Source Data file.

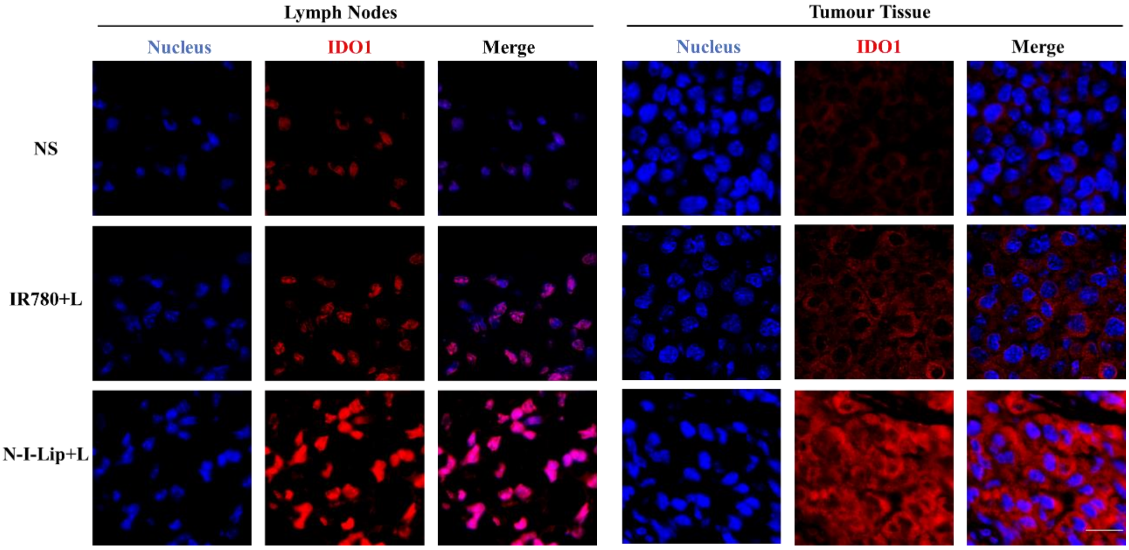

Supplementary Fig. 8. Evaluation of IDO1 expression both in the LNs and tumours after photothermal therapy. Immunohistochemical imaging of the LNs and tumours in the NS group, free IR780+L group and N-I-Lip+L group. The mice were treated with the corresponding formulation every 4 days and irradiated with an 808 nm laser within 5 min after intravenous formulation administration. After 16 days, the LNs and tumours were removed for immunohistochemical imaging (n = 3 biologically independent experiments). The scale bar represents 20 μm. IDO1: indoleamine 2,3-dioxygenase-1.

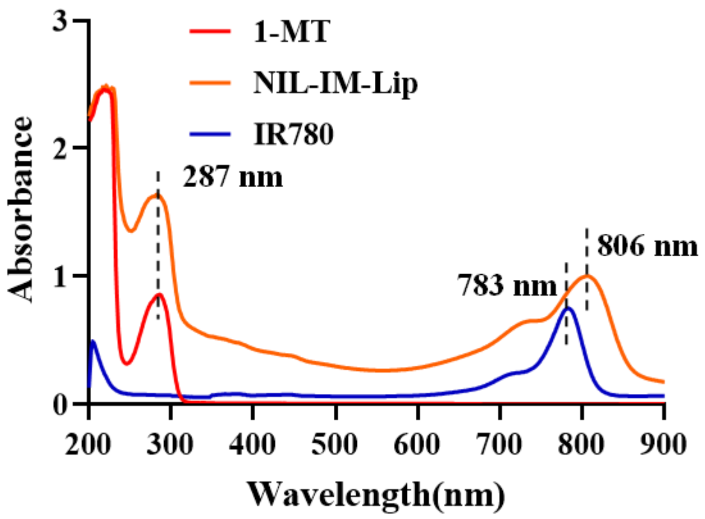

Supplementary Fig. 9. The UV-Vis absorption spectra of free 1-MT, free IR780 and NIL-IM-Lip.

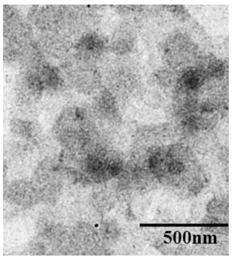

Supplementary Fig. 10. The TEM morphology of NIL-IM-Lip after irradiation of 808 nm laser (1.0 W/cm^2, 5 min) (n = 3 biologically independent experiments). TEM: Transmission Electron Microscope

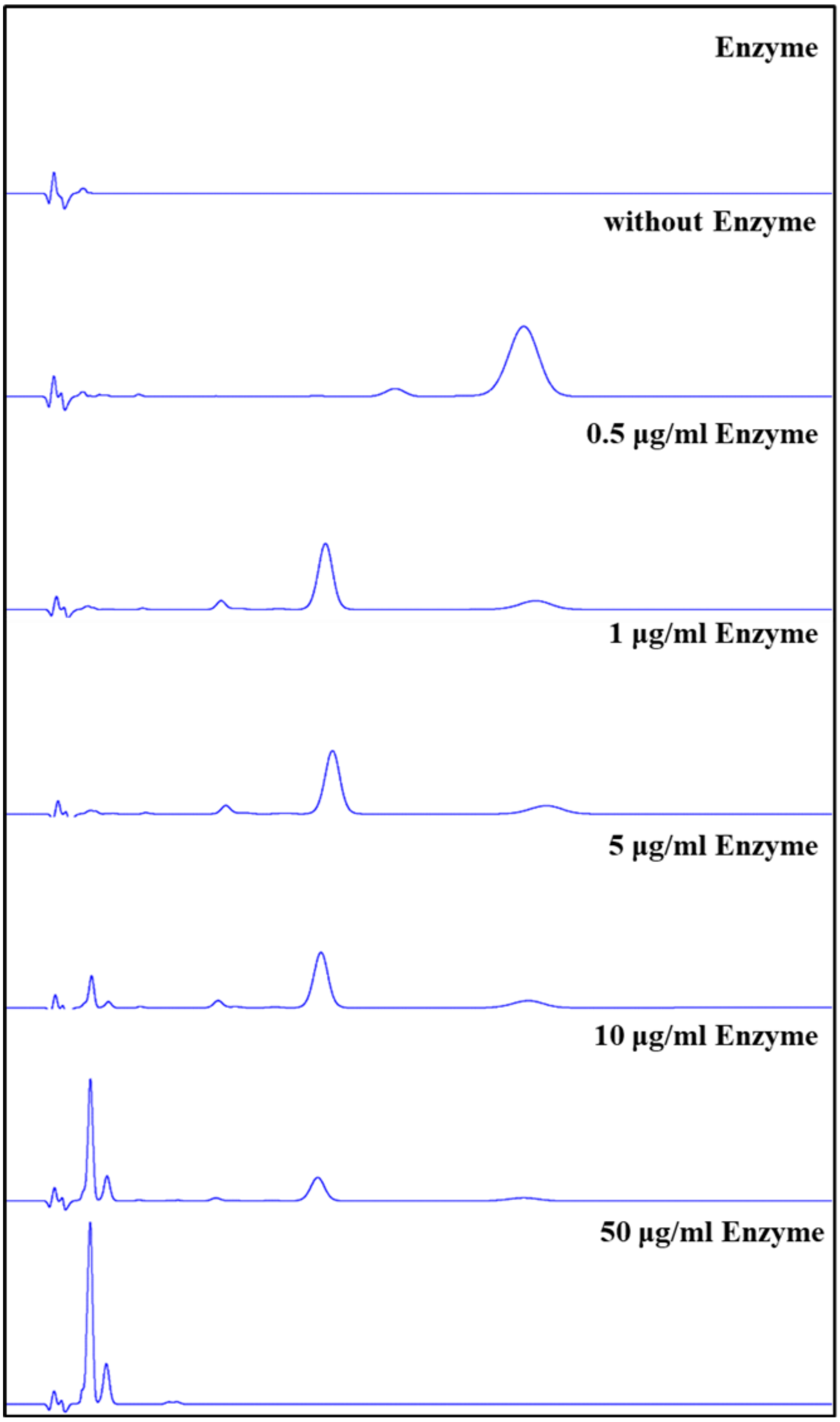

Supplementary Fig. 11. The HPLC chromatograms of MMP2 enzyme alone and The HPLC chromatograms of 1.0 mg/mL MMP2 sensitive peptide after incubated with different concentrations MMP2 enzyme.

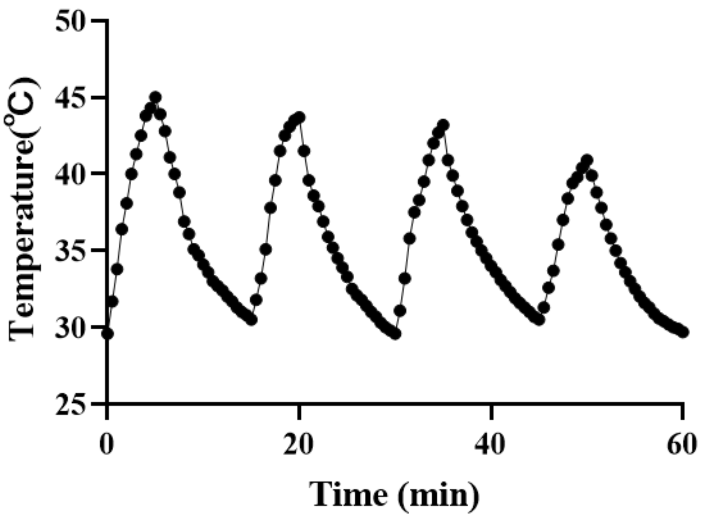

Supplementary Fig. 12. The free IR780 showed good photothermal stability. The temperature variation curves of free IR780 were recorded under four cycles of the laser (808 nm) on/off.

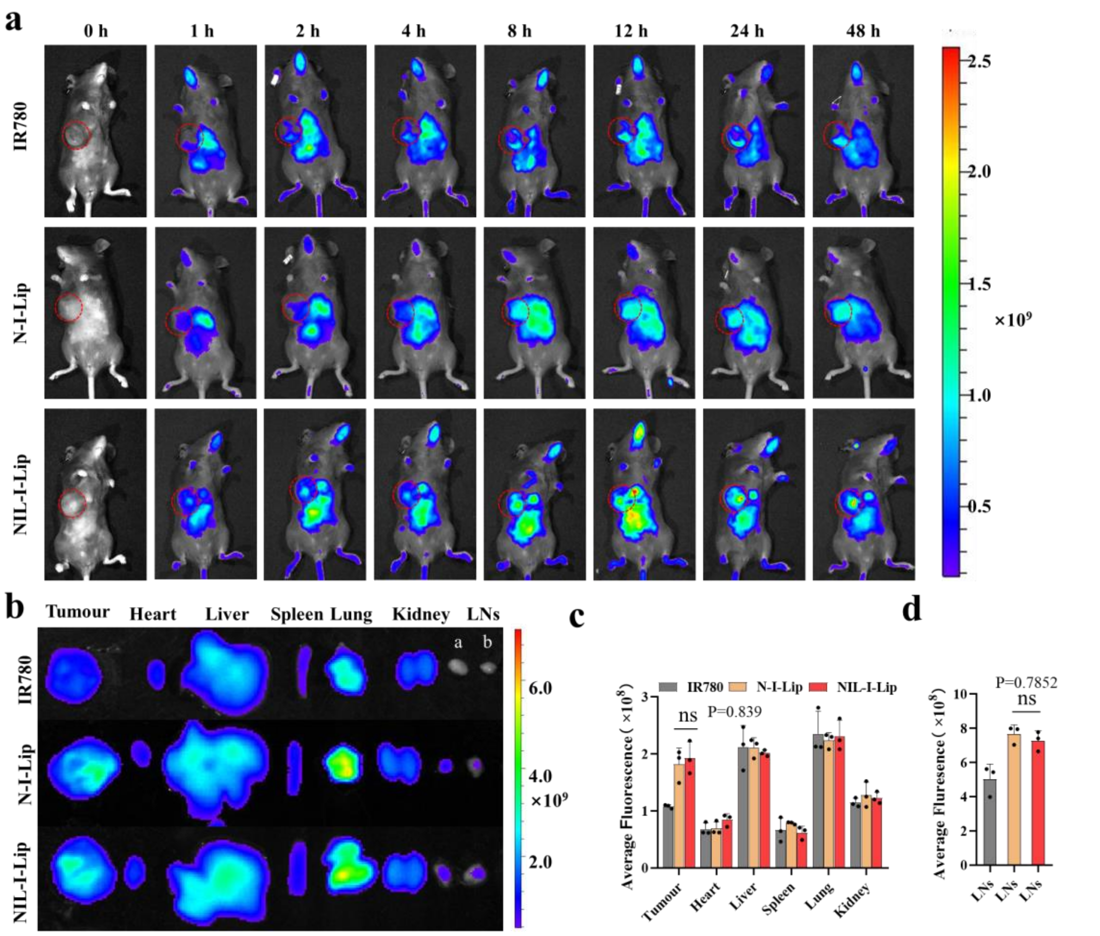

Supplementary Fig. 13. N-I-Lip and NIL-I-Lip showed similar tumour and LNs accumulation. a, *In vivo* biodistribution imaging of the mice after injection of IR780, N-I-Lip and NIL-I-Lip (*i.v.*) (n = 3 biologically independent animals per group). **b,** *Ex vivo* imaging of the tumour, main organs (including heart, liver, spleen, lung, kidneys) and LNs at 48 h (n = 3 biologically independent animals per group). **c,** Average fluorescence intensities of the tumour, main organs at 48 h (n = 3 biologically independent experiments). **d,** Average fluorescence intensities of the LNs at 48 h (n = 3 biologically independent experiments). Data are presented as mean values ± SD. Statistical significance was calculated by one-way ANOVA analysis of variance with Tukey's post hoc test. ns means not significant difference. P>0.05. Source data are provided as a Source Data file.

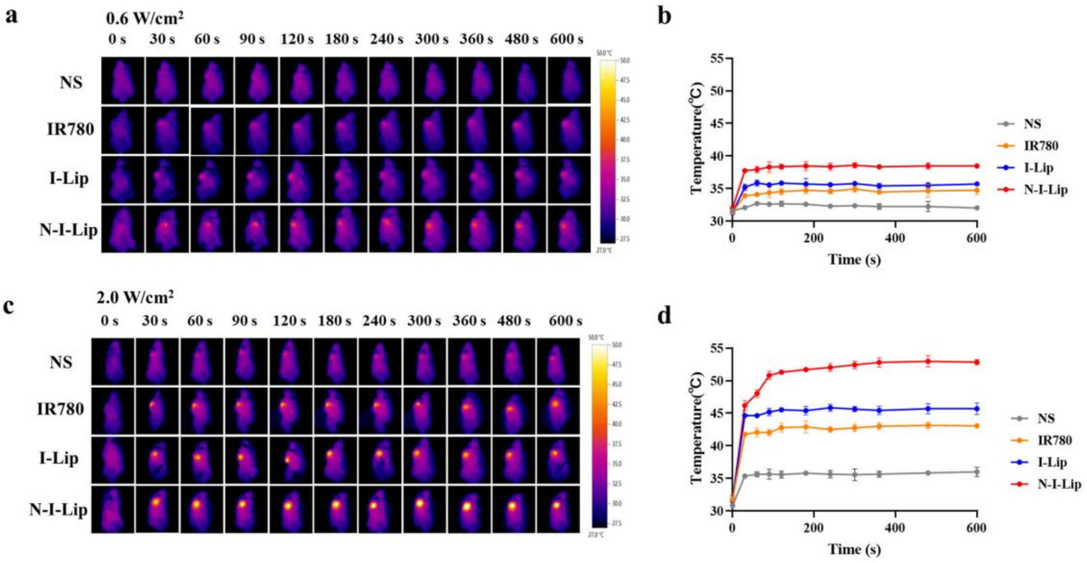

Supplementary Fig. 14. The Infrared thermal imaging and the temperature profile of tumour under 0.6 and 1.0 W/cm^2 laser. a, Infrared thermal imaging of B10F10 bearing mice treated with NS, IR780, I-Lip and N-I-Lip under 0.6 W/cm^2 laser (808 nm). **b,** Temperature profile of tumour on B10F10 bearing mice treated with NS, IR780, I-Lip and N-I-Lip under 0.6 W/cm^2 laser (808 nm) (n = 3 biologically independent experiments). **c,** Infrared thermal imaging of B10F10 bearing mice treated with NS, IR780, I-Lip and N-I-Lip under 2.0 W/cm^2 laser (808 nm). **d,** Temperature profile of tumour on B10F10 bearing mice treated with NS, IR780, I-Lip and N-I-Lip under 2.0 W/cm^2 laser (808 nm) (n = 3 biologically independent experiments). Data are presented as mean values ± SD. Source data are provided as a Source Data file.

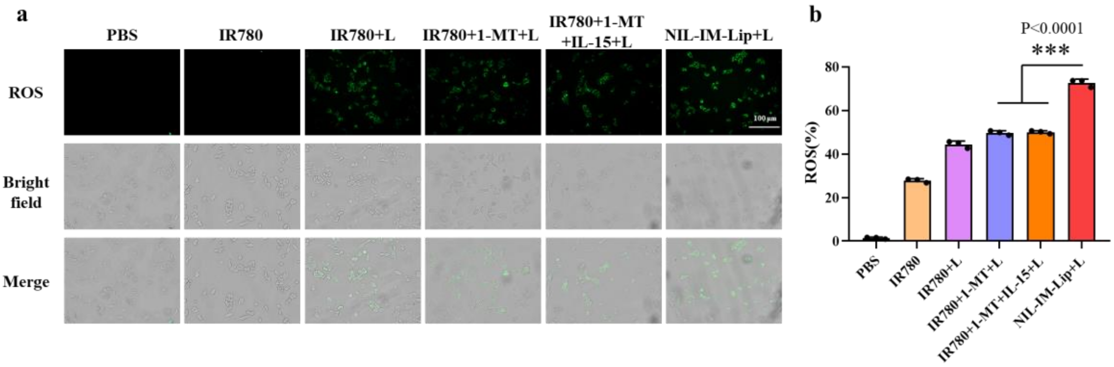

Supplementary Fig. 15. NIL-IM-Lip+L induced stronger ROS generation in *vitro*. **a,** The fluorescence microscopy images of ROS generation in B16F10 cells. Scale bar: 100 µm. **b,** The flow cytometric analysis of ROS generation in B16F10 cells (n = 3 biologically independent experiments). Data are presented as mean values ± SD. Statistical significance was calculated by one-way ANOVA analysis of variance with Tukey's post hoc test. ***$P < 0.001$. Source data are provided as a Source Data file.

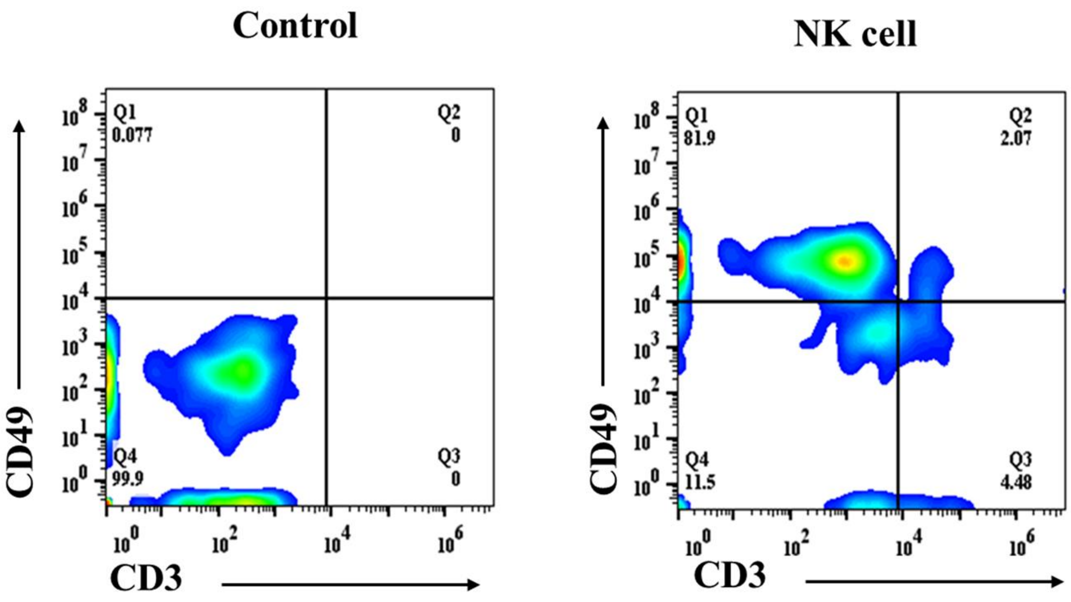

Supplementary Fig. 16. The purity of NK cells separated by MACS method. The mice were sacrificed and the NK cells were separated from spleen of female C57BL/6 mice by MACS method. The obtained NK cells were marked as $CD3^-CD49^+$. MACS: Magnetic activated cell sorting.

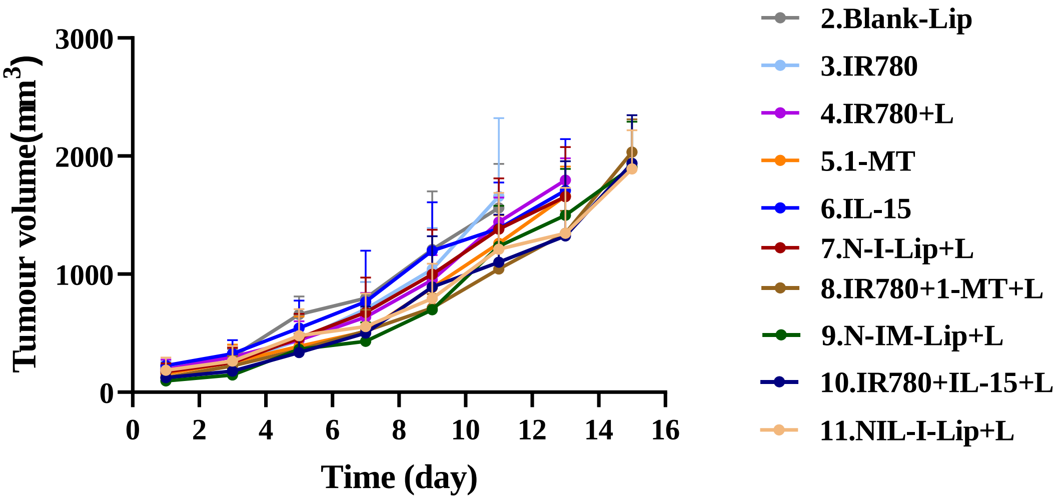

Supplementary Fig. 17. Tumour volume curves of different formulation-treated B16F10 tumour-bearing C57BL/6 mice (n = 6 biologically independent animals per group). Data are presented as mean values ± SD. Source data are provided as a Source Data file.

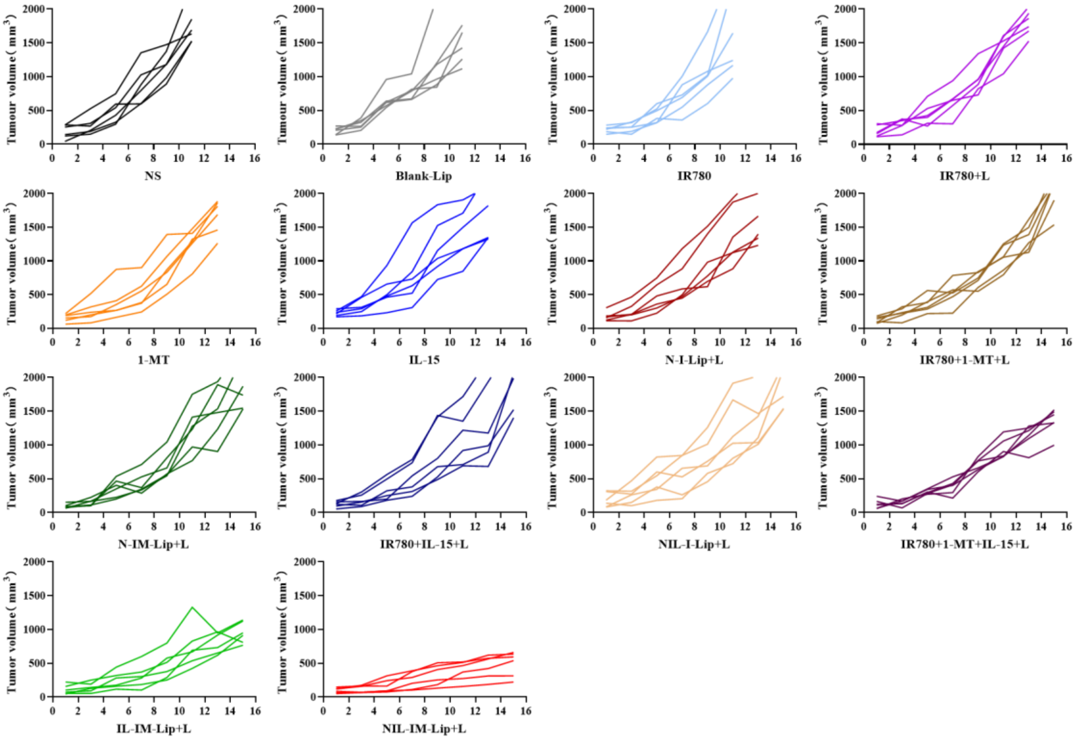

Supplementary Fig. 18. The individual tumour growth curves of B16F10-bearing C57BL/6 mice after intravenous injection with different formulations, including: NS, Blank-Lip, IR780, IR780+L, 1-MT, IL-15, N-I-Lip+L, IR780+1-MT+L, N-IM-Lip+L, IR780+IL-15+L, NIL-I-Lip+L, IR780+1-MT+IL-15+L, NIL-IM-Lip+L (n = 6 biologically independent animals per group).

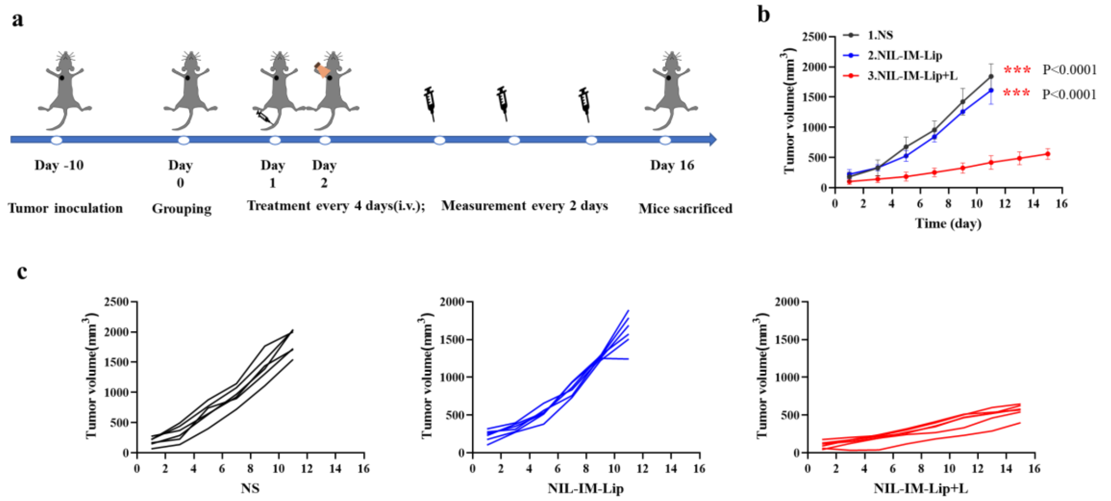

Supplementary Fig. 19. NIL-IM-Lip treatment with laser irradiation showed enhanced antitumour efficacy compared with NIL-IM-Lip without laser irradiation. a, The antitumour B16F10 tumour-bearing C57BL/6 mouse experiment schedule. **b,** Tumour volume curves of different formulation-treated B16F10 tumour-bearing C57BL/6 mice (n = 6 biologically independent animals per group). **c,** Individual tumour volume curves of different formulation-treated B16F10 tumour-bearing C57BL/6 mice (n = 6 biologically independent animals per group). Data are presented as mean values ± SD. Statistical significance was calculated by one-way ANOVA analysis of variance with Tukey's post hoc test. ***$P < 0.001$. Source data are provided as a Source Data file.

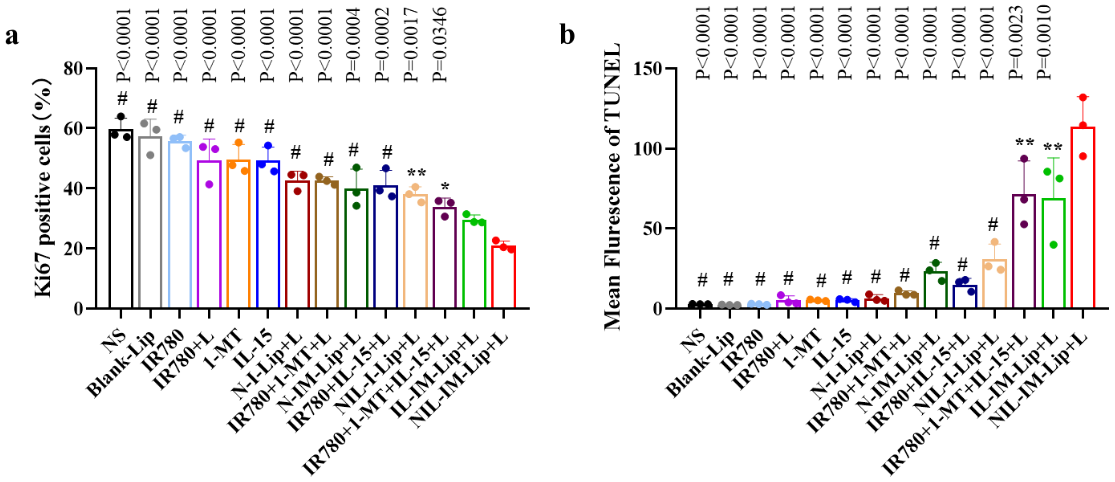

Supplementary Fig. 20. Quantification of (a) Ki67 and (b) TUNEL staining assays of tumour tissues (n = 3 biologically independent experiments)**.** Data are presented as mean values ± SD. Statistical significance was calculated by one-way ANOVA analysis of variance with Tukey's post hoc test. *P < 0.05, **P < 0.01, # P < 0.001. Source data are provided as a Source Data file.

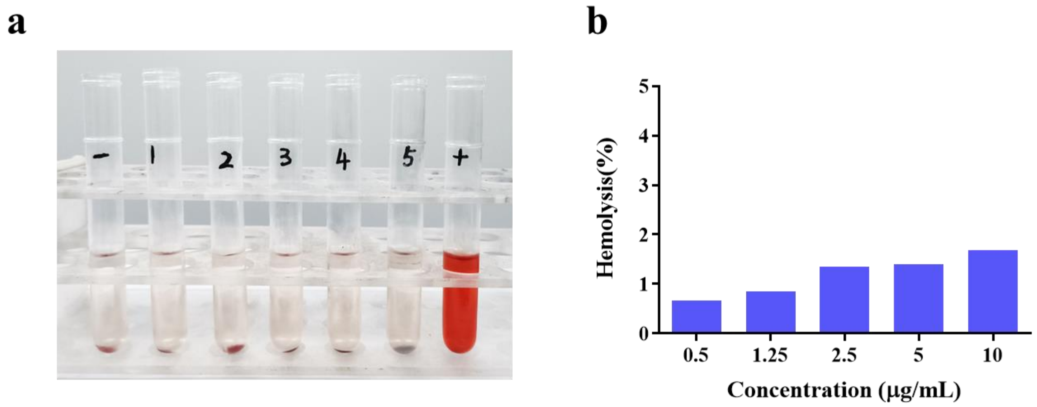

Supplementary Fig. 21. The evaluation of hemolysis assay of NIL-IM-Lip *in vitro.*

a, The hemolysis photograph of NIL-IM-Lip. **b,** The hemolysis rates of different concentrations NIL-IM-Lip.

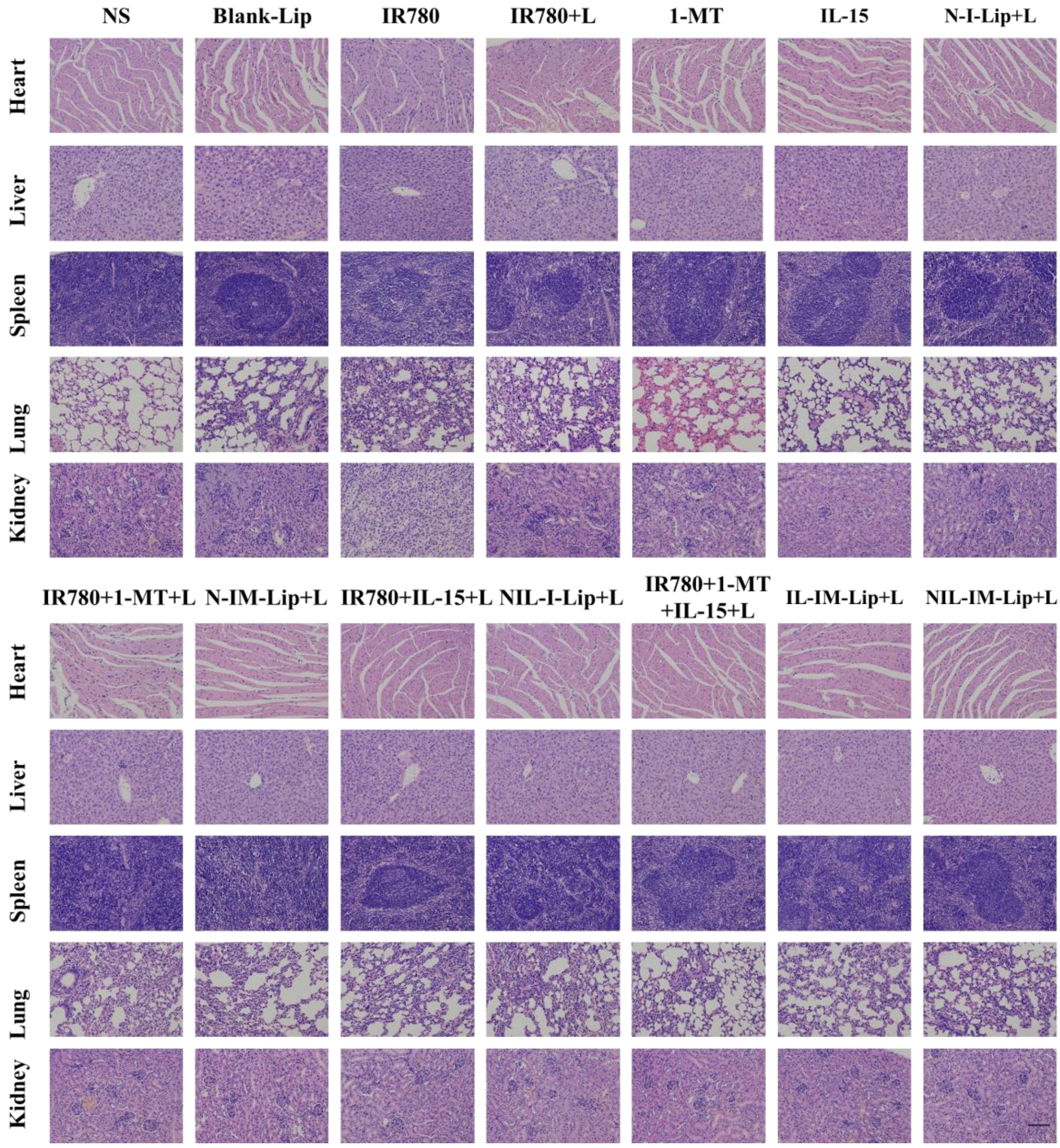

Supplementary Fig. 22. The H&E staining of heart, liver, spleen, lung and kidneys from B16F10 bearing mice with different formulations treatment (n = 3 biologically independent experiments). Scale bar:100 μm.

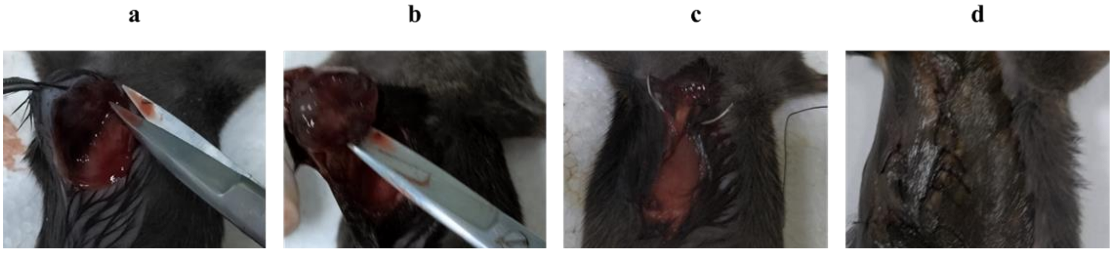

Supplementary Fig. 23. The establishment process of postsurgical model. a, Anesthesia and surgery. **b,** Remove 90% tumour. **c,** Suture the wound. **d,** Disinfection the wound.

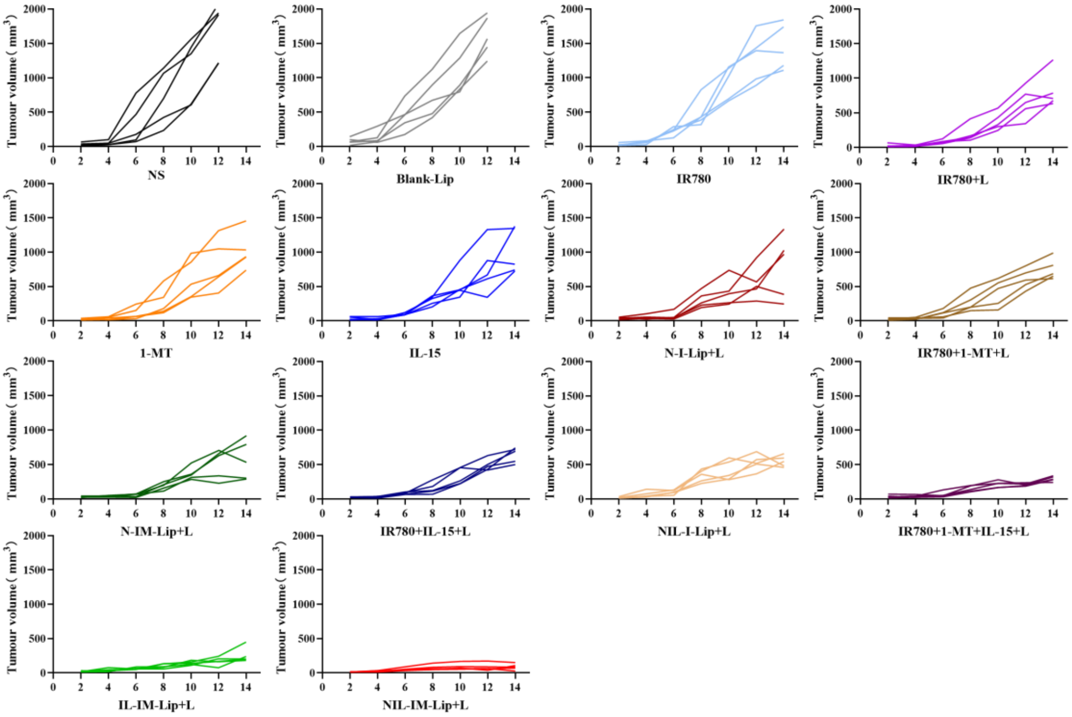

Supplementary Fig. 24. The individual tumour growth curves of B16F10-bearing C57BL/6 mice in postsurgical model. The mice were intravenously injected with different formulations, including: NS, Blank-Lip, IR780, IR780+L, 1-MT, IL-15, N-I-Lip+L, IR780+1-MT+L, N-IM-Lip+L, IR780+IL-15+L, NIL-I-Lip+L, IR780+1-MT+IL-15+L, NIL-IM-Lip+L (n = 5 biologically independent animals per group).

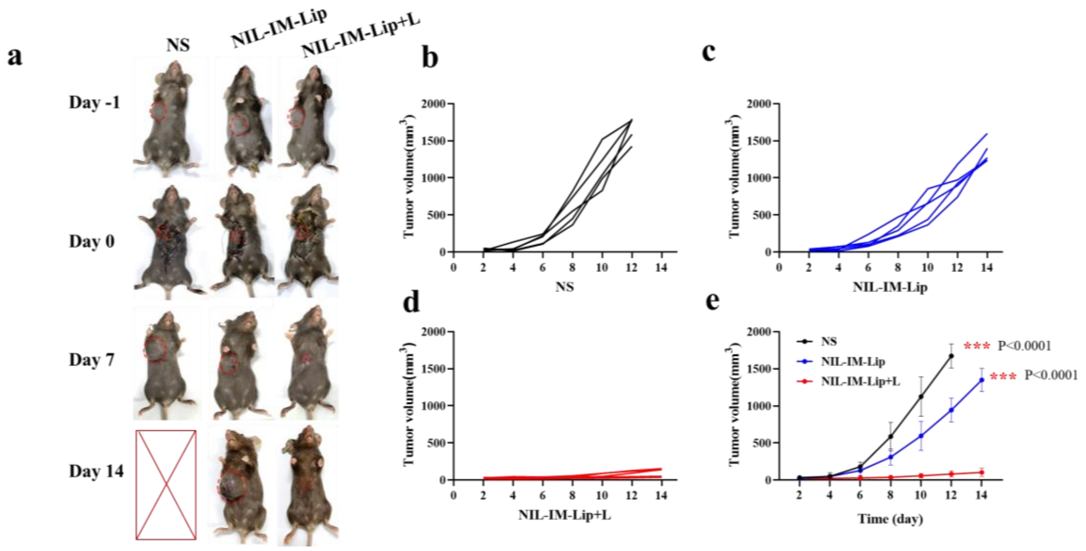

Supplementary Fig. 25. NIL-IM-Lip with laser showed enhanced antitumour efficacy than NIL-IM-Lip without laser in postsurgical model. a. The photograph of mice on Day-1, 0,7 and 14. **b-d,** The individual tumour volume curve of mice treated with NS, NIL-IM-Lip, NIL-IM-Lip+L. **e,** The tumour volume curve of NS, NIL-IM-Lip and NIL-IM-Lip+L groups (n = 5 biologically independent animals per group). Data are presented as mean values ± SD. Statistical significances were calculated by one-way ANOVA analysis of variance with Tukey's post hoc test. ***$P < 0.001$. Source data are provided as a Source Data file.

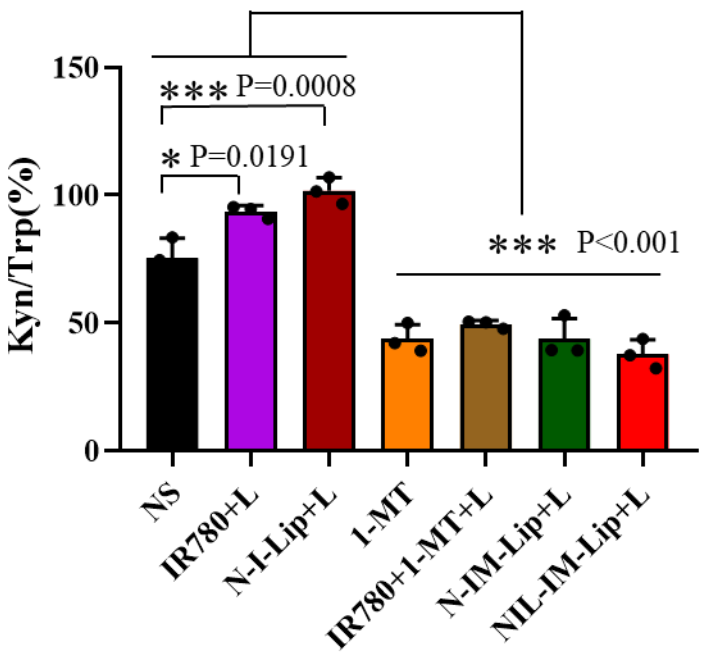

Supplementary Fig. 26. The ratio of Kyn and Trp in tumour tissues obtained from B16F10-bearing mice were determined by HPLC method (n = 3 biologically independent experiments**).** HPLC: High performance liquid chromatography. Data are presented as mean values ± SD. Statistical significances were calculated by one-way ANOVA analysis of variance with Tukey's post hoc test. $*P < 0.05$, $***P < 0.001$. Source data are provided as a Source Data file.

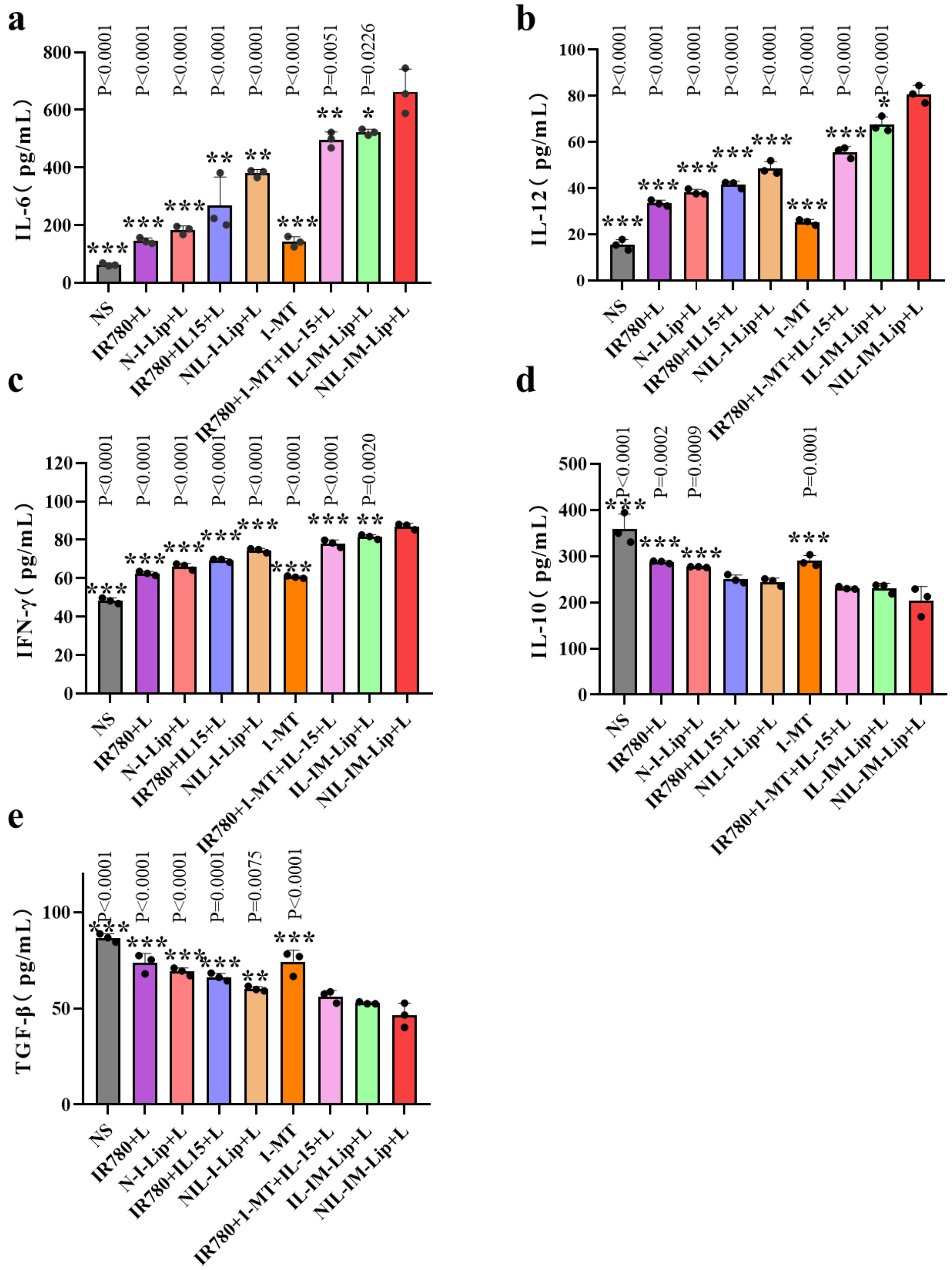

Supplementary Fig. 27. The level of cytokines in tumour tissues. a, The level of IL-6, **b,** IL-12, **c,** IFN-γ, **d,** IL-10, **e,** TGF-β in tumour tissues (n = 3 biologically independent experiments). Data are presented as mean values ± SD. Statistical significances were calculated by one-way analysis of variance with Tukey's post hoc test. *$P < 0.05$, **$P < 0.01$, ***$P < 0.001$.

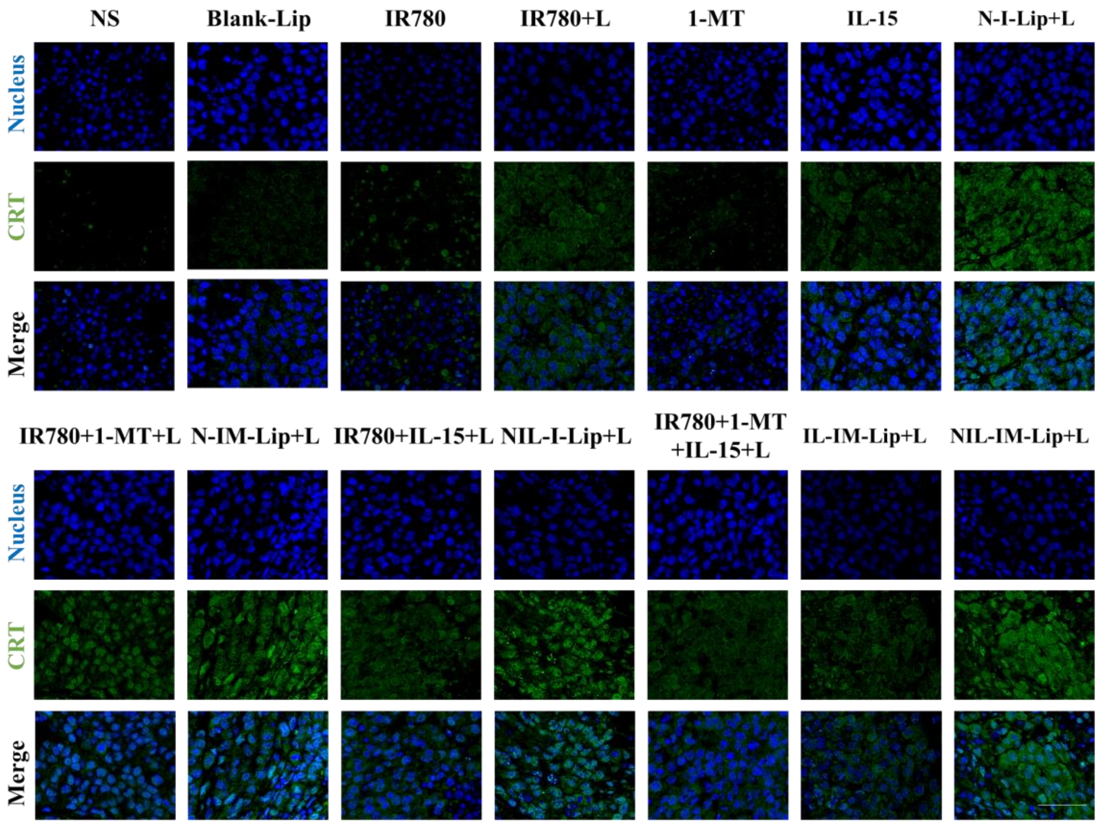

Supplementary Fig. 28. Evaluation of CRT exposure in tumour tissues. Immunohistochemical imaging of CRT in tumours with treatment of NS, Blank-Lip, IR780, IR780+L, 1-MT, IL-15, N-I-Lip+L, IR780+1-MT+L, N-IM-Lip+L, IR780+IL-15+L, NIL-I-Lip+L, IR780+1-MT+IL-15+L, NIL-IM-Lip+L (n = 3 biologically independent experiments). Scale bar: 50 μm.

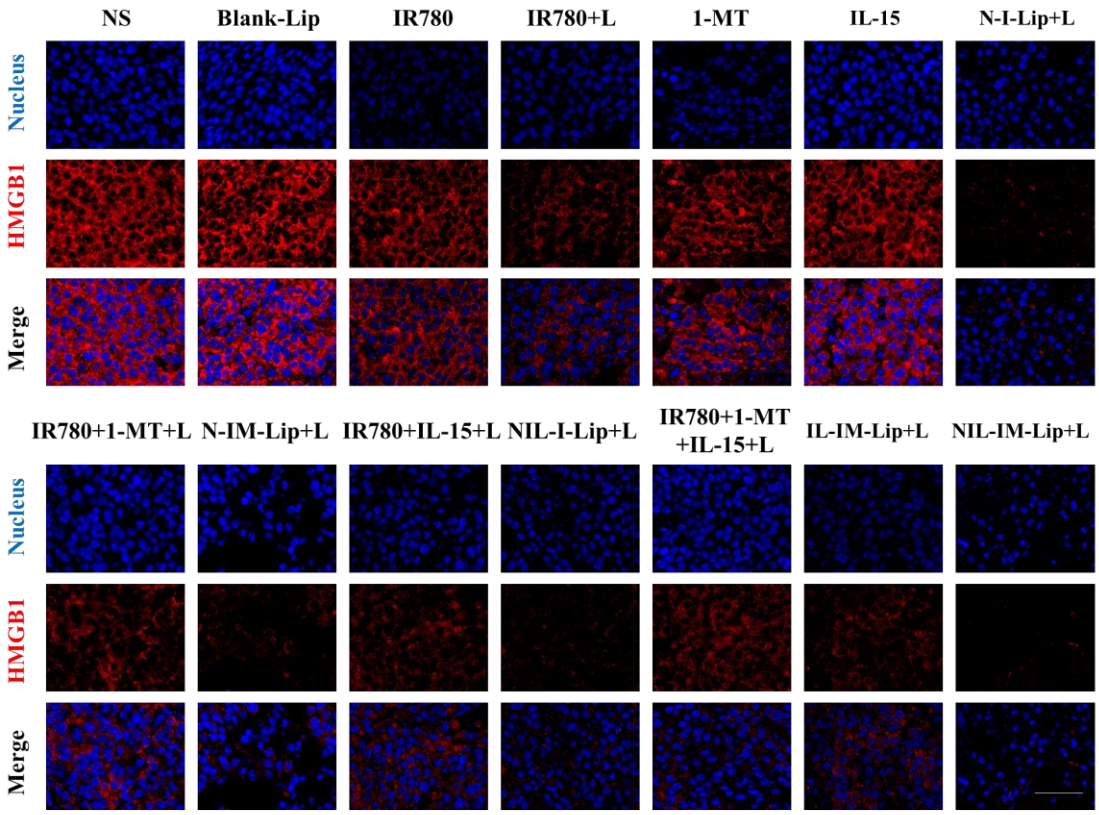

Supplementary Fig. 29. Evaluation of HMGB1 release in tumour tissues. Immunohistochemical imaging of HMGB1 release in tumours with treatment of NS, Blank-Lip, IR780, IR780+L, 1-MT, IL-15, N-I-Lip+L, IR780+1-MT+L, N-IM-Lip+L, IR780+IL-15+L, NIL-I-Lip+L, IR780+1-MT+IL-15+L, NIL-IM-Lip+L ($n = 3$ biologically independent experiments). Scale bar: 50 μm.

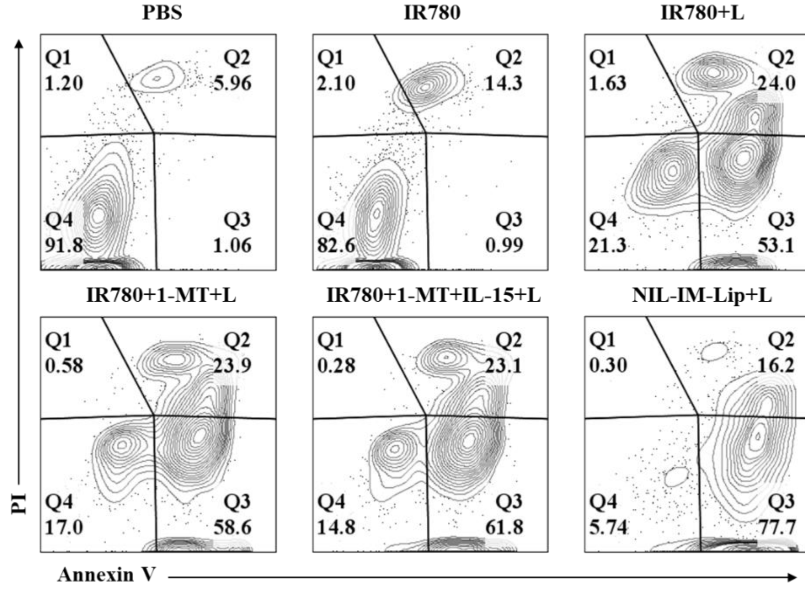

Supplementary Fig. 30. Flow cytometric apoptosis analysis of CT26 cells after treatment with PBS, IR780, IR780+L, IR780+1-MT+L, IR780+1-MT+IL-15+L and NIL-IM-Lip+L. The IR780+L, IR780+1-MT+L, IR780+1-MT+IL-15+L and NIL-IM-Lip+L groups were irradiated with an 808 nm laser for 5 min (1.0 W/cm^2).

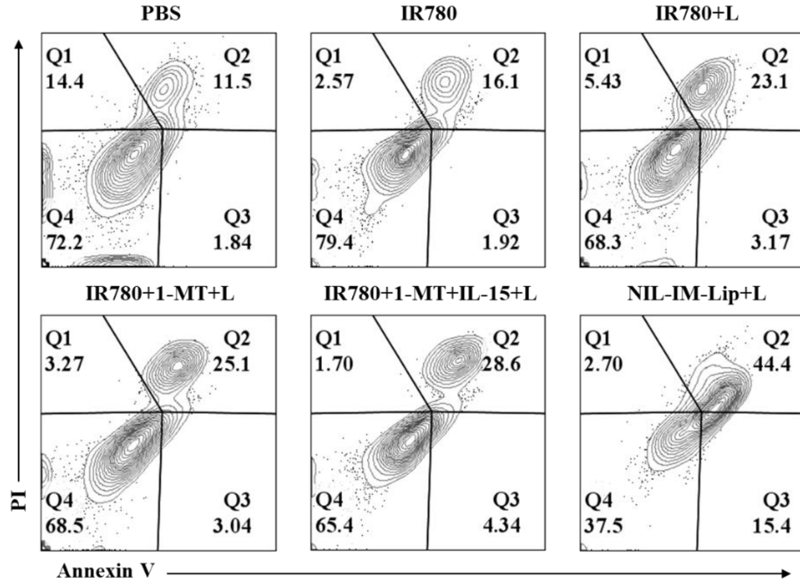

Supplementary Fig. 31. Flow cytometric apoptosis analysis of MC38 cells after treatment with PBS, IR780, IR780+L, IR780+1-MT+L, IR780+1-MT+IL-15+L and NIL-IM-Lip+L. The IR780+L, IR780+1-MT+L, IR780+1-MT+IL-15+L and NIL-IM-Lip+L groups were irradiated with an 808 nm laser for 5 min (1.0 W/cm^2).

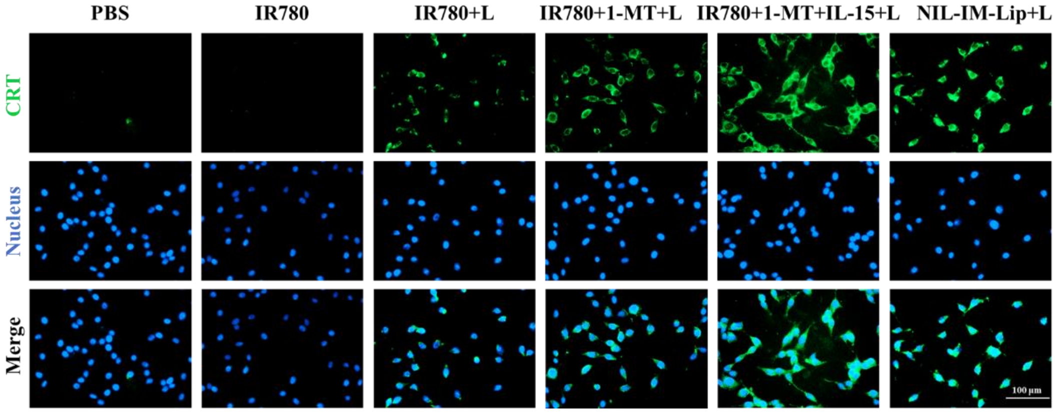

Supplementary Fig. 32. Fluorescence microscopy images of CRT exposure to CT26 cells. The IR780+L, IR780+1-MT+L, IR780+1-MT+IL-15+L and NIL-IM-Lip+L groups were irradiated with an 808 nm laser for 5 min (1.0 W/cm^2) (n = 3 biologically independent experiments). Scale bar: 100 μm.

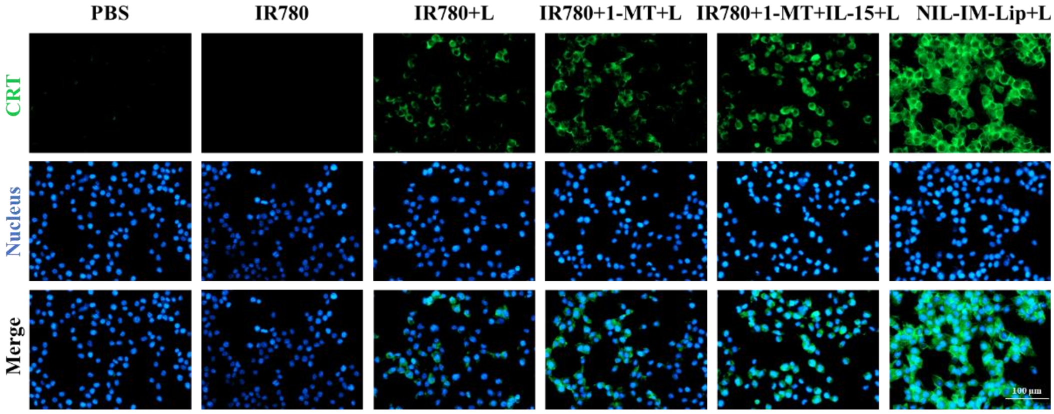

Supplementary Fig. 33. Fluorescence microscopy images of CRT exposure to MC38 cells. The IR780+L, IR780+1-MT+L, IR780+1-MT+IL-15+L and NIL-IM-Lip+L groups were irradiated with an 808 nm laser for 5 min (1.0 W/cm^2) (n = 3 biologically independent experiments). Scale bar: 100 μm.

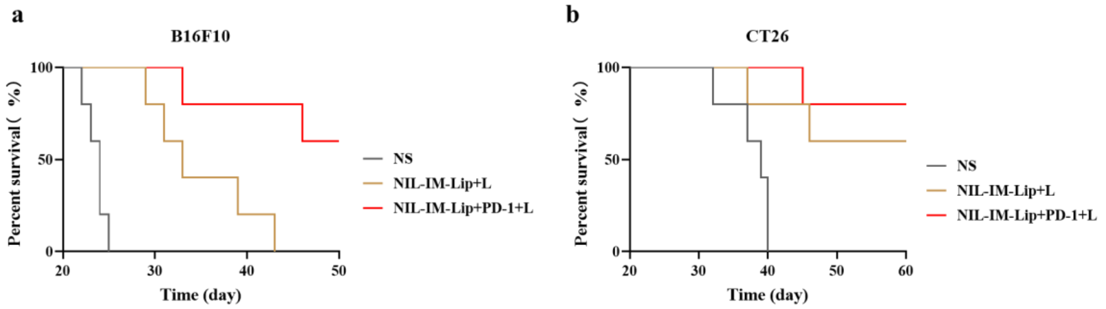

Supplementary Fig. 34. The percent survival of NS group, NIL-IM-Lip+L group, NIL-IM-Lip+PD-1+L group on both B16F10 model and CT26 model (n = 5 biologically independent animals per group).

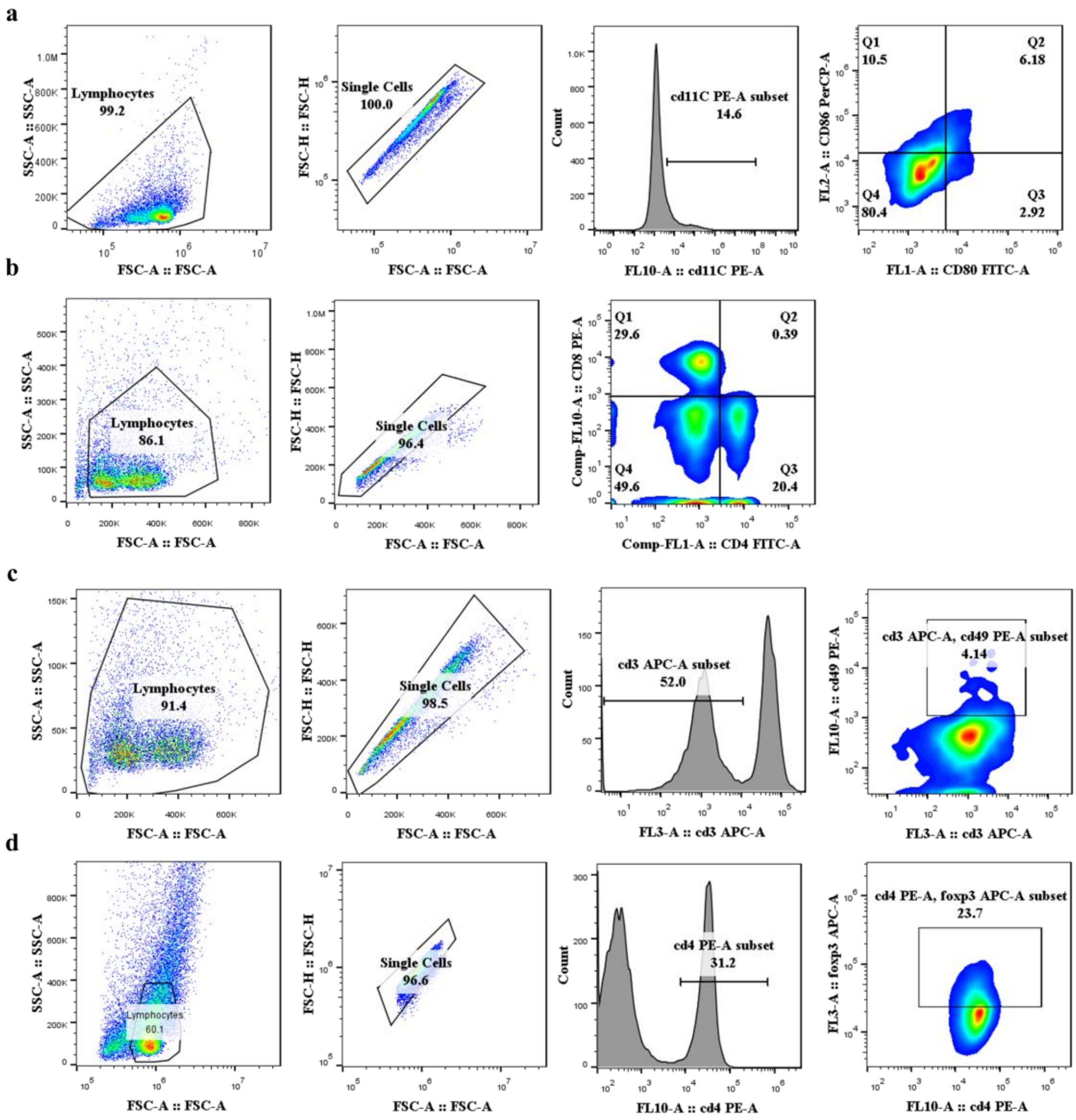

Supplementary Fig. 35. Gating strategies for immune cells in LNs presented on Fig. 7i-p and Fig. 8h,i,p,r. a, Gating strategy for DC cells (CD11c^{+}CD80^{+}CD86^{+}). **b,** Gating strategy for CD4^{+}T cells and CD8^{+}T cells. **c,** Gating strategy for NK cells (CD3^{-}CD49b^{+}). **d,** Gating strategy for Tregs (CD4^{+}Foxp3^{+}).

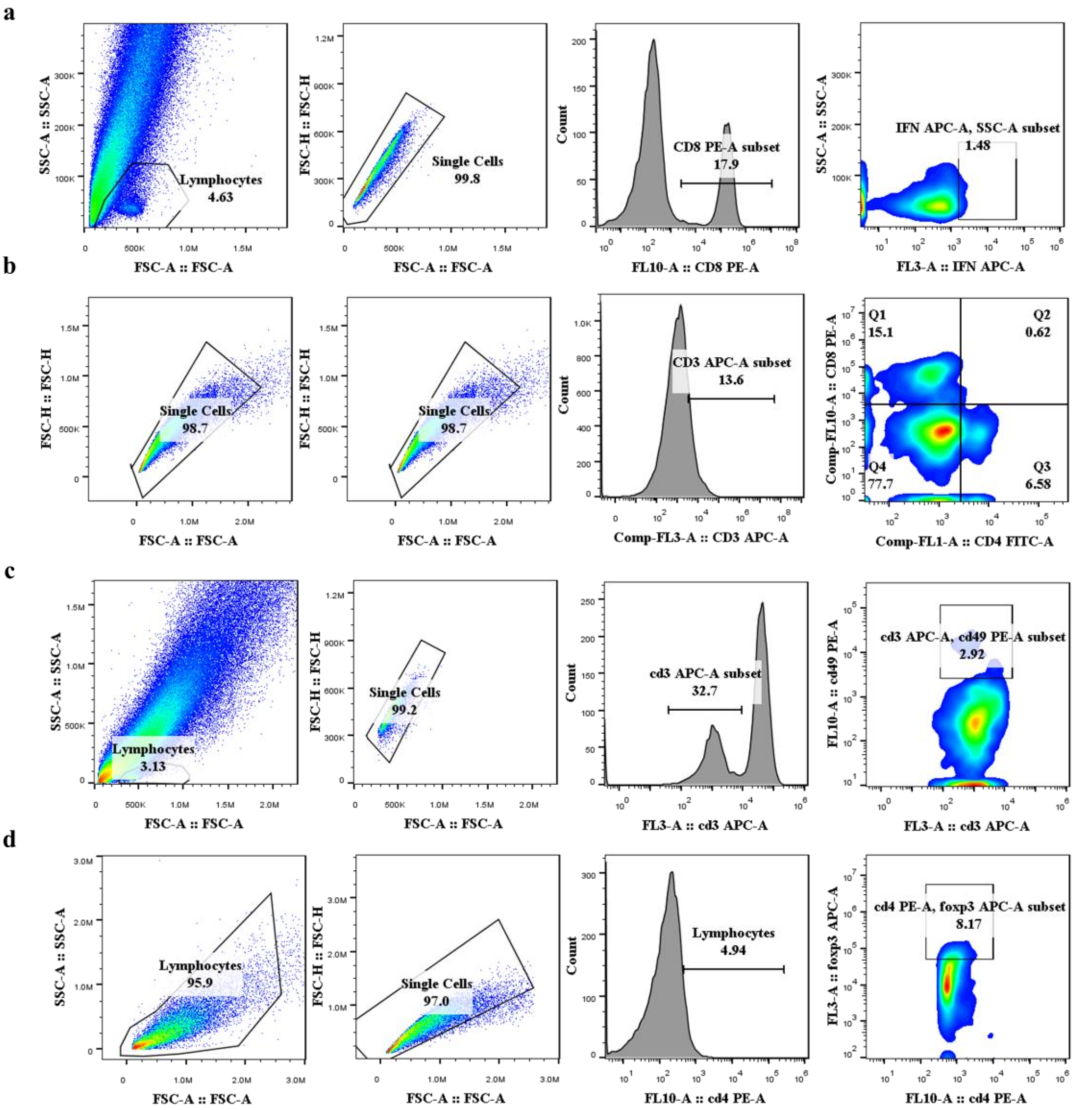

Supplementary Fig. 36. Gating strategies for immune cells in tumours presented on Fig. 7a-f and Fig. 8f,g,o,q. a, Gating strategy for CTLs ($CD8^+IFN\text{-}\gamma^+$). **b,** Gating strategy for $CD4^+T$ ($CD3^+$ $CD4^+$) cells and $CD8^+T$ ($CD3^+$ $CD8^+$) cells. **c,** Gating strategy for NK cells ($CD3^-CD49b^+$). **d,** Gating strategy for Treg cells ($CD4^+Foxp3^+$).

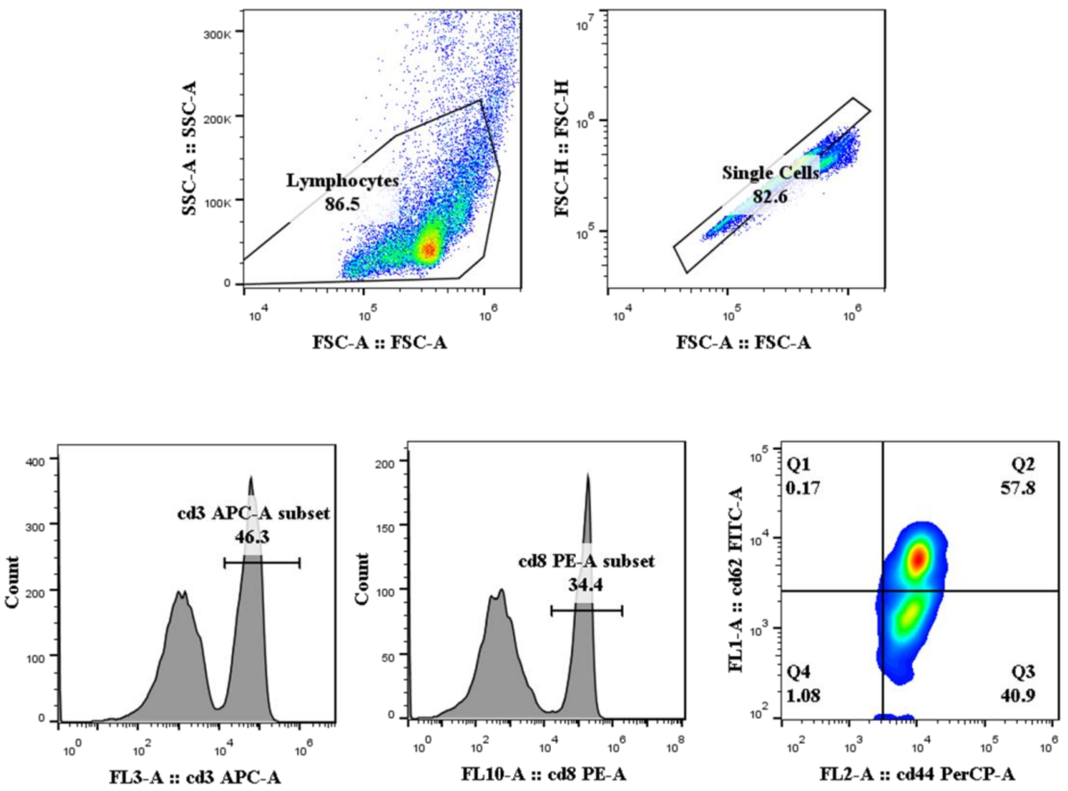

Supplementary Fig. 37. Gating strategies for memory T cells ($CD3^{+}CD8^{+}CD44^{+}CD62L^{-}$) in spleens presented on Fig. 6k,l.

Supplementary Table 1. The characteristic of NIL-IM-Lip with different particle size and mass ratio.

	DPPC:Chol=8:1		N-I-Lip-S	
	N-I-Lip-L	N-I-Lip-S	DPPC:Chol=2:1	DPPC:Chol=16:1
Size (nm)	134.9±2.7	63.6±4.5	63.3±4.7	69.3±0.8
PDI	0.181±0.06	0.211±0.05	0.286±0.03	0.200±0.05

Supplementary Table 2. The characteristic of NIL-IM-Lip

Size (nm)	PDI	Zeta (mV)	1-MT EE%	1-MT DL%	IR780 EE%	IR780 DL%
66.9±2.2	0.267±0.01	-2.0±1.0	30.5±3.2	3.0±0.3	81.8±2.8	0.64±0.02
