# Supplementary material for: Temperature sensitive liposome based cancer nanomedicine enables tumour lymph node immune microenvironment remodelling
Source: Nat Commun. 2023 Apr 19;14:2248. doi: 10.1038/s41467-023-38014-6 (PMC10115817; doi:10.1038/s41467-023-38014-6)
Supplement: Supplementary file 3 — Reporting Summary [file 41467_2023_38014_MOESM3_ESM.pdf]

nature portfolio

Corresponding author(s): Yongjun Liu,Na Zhang

Last updated by author(s): Mar 23, 2023

Reporting Summary

Nature Portfolio wishes to improve the reproducibility of the work that we publish. This form provides structure for consistency and transparency in reporting. For further information on Nature Portfolio policies, see our Editorial Policies and the Editorial Policy Checklist.

Statistics

For all statistical analyses, confirm that the following items are present in the figure legend, table legend, main text, or Methods section.

n/a	Confirmed	
☐	☒	The exact sample size (*n*) for each experimental group/condition, given as a discrete number and unit of measurement
☐	☒	A statement on whether measurements were taken from distinct samples or whether the same sample was measured repeatedly
☐	☒	The statistical test(s) used AND whether they are one- or two-sided<br>*Only common tests should be described solely by name; describe more complex techniques in the Methods section.*
☐	☒	A description of all covariates tested
☐	☒	A description of any assumptions or corrections, such as tests of normality and adjustment for multiple comparisons
☐	☒	A full description of the statistical parameters including central tendency (e.g. means) or other basic estimates (e.g. regression coefficient) AND variation (e.g. standard deviation) or associated estimates of uncertainty (e.g. confidence intervals)
☐	☒	For null hypothesis testing, the test statistic (e.g. *F*, *t*, *r*) with confidence intervals, effect sizes, degrees of freedom and *P* value noted<br>*Give P values as exact values whenever suitable.*
☒	☐	For Bayesian analysis, information on the choice of priors and Markov chain Monte Carlo settings
☒	☐	For hierarchical and complex designs, identification of the appropriate level for tests and full reporting of outcomes
☒	☐	Estimates of effect sizes (e.g. Cohen's *d*, Pearson's *r*), indicating how they were calculated

Our web collection on statistics for biologists contains articles on many of the points above.

Software and code

Policy information about availability of computer code

Data collection	BD Accuri C6 Plus, Beckman Coulter CytoFLEX S, Zeiss LSM 900 with AiryScan 2, Perkin Elmer IVIS Spectrum, DLS/zeta (NanoZS90,Malvern)
Data analysis	FlowJo 10 was used for flow cytometry data analysis; GraphPad Prism 8.0.1 and Microsoft Excel 2019 were used for statistical analysis; The histological section images were analyzed using CaseViewer 2.4.

For manuscripts utilizing custom algorithms or software that are central to the research but not yet described in published literature, software must be made available to editors and reviewers. We strongly encourage code deposition in a community repository (e.g. GitHub). See the Nature Portfolio guidelines for submitting code & software for further information.

Data

Policy information about availability of data

All manuscripts must include a data availability statement. This statement should provide the following information, where applicable:

- Accession codes, unique identifiers, or web links for publicly available datasets
- A description of any restrictions on data availability
- For clinical datasets or third party data, please ensure that the statement adheres to our policy

The data supporting the findings of this study are available within this article and its Supplementary Information, source data file. Source data are provided with this paper.

Human research participants

Policy information about studies involving human research participants and Sex and Gender in Research.

Reporting on sex and gender	*Use the terms sex (biological attribute) and gender (shaped by social and cultural circumstances) carefully in order to avoid confusing both terms. Indicate if findings apply to only one sex or gender; describe whether sex and gender were considered in study design whether sex and/or gender was determined based on self-reporting or assigned and methods used. Provide in the source data disaggregated sex and gender data where this information has been collected, and consent has been obtained for sharing of individual-level data; provide overall numbers in this Reporting Summary. Please state if this information has not been collected. Report sex- and gender-based analyses where performed, justify reasons for lack of sex- and gender-based analysis.*
Population characteristics	*Describe the covariate-relevant population characteristics of the human research participants (e.g. age, genotypic information, past and current diagnosis and treatment categories). If you filled out the behavioural & social sciences study design questions and have nothing to add here, write "See above."*
Recruitment	*Describe how participants were recruited. Outline any potential self-selection bias or other biases that may be present and how these are likely to impact results.*
Ethics oversight	*Identify the organization(s) that approved the study protocol.*

Note that full information on the approval of the study protocol must also be provided in the manuscript.

Field-specific reporting

Please select the one below that is the best fit for your research. If you are not sure, read the appropriate sections before making your selection.

☒ Life sciences ☐ Behavioural & social sciences ☐ Ecological, evolutionary & environmental sciences

For a reference copy of the document with all sections, see nature.com/documents/nr-reporting-summary-flat.pdf

Life sciences study design

All studies must disclose on these points even when the disclosure is negative.

Sample size	Sample size are provided in the figure legends for each experiment and reasonable sample sizes were chosen to ensure they are sufficient for statistical comparison between different groups. For in vitro experiments and analysis, a sample size of 3 was used to detect a significant difference between different groups. For analysing the lymph node accumulation, 3 mice of each group were used for in vivo imaging and then the mice were euthanized for ex vivo imaging. The in vivo efficacy studies of the NIL-IM-Lip+L were performed with 6 mice per group. For the combination therapy of the NIL-IL-Lip with anti-PD-1 mAb, 5 mice per group were used.Details regarding sample size of all experiments are provided in the Methods section and figure legends. Samples sizes for in vivo experiments were chosen empiricallbased the antitumour research and considering of the approval from ShandongUniversity's Institutional Animal Care and Use Committee. Sample sizes for in vitro experiments were also chosen empirically based upon preliminary experiments to achieve statistical significance.
Data exclusions	No data was excluded from the analyses.
Replication	Experiments were replicated independently for at least 3 times. The number of replicates is detailed in the caption of each figures in the main manuscript and supplementary information files. Experiments were repeated and experimental findings were reproducible.
Randomization	In vitro experiments, the cultured cells were randomly assigned to experimental groups. In vivo experiments, the mice were randomized into different groups before treatment.
Blinding	No formal blinding was used due to all data were acquired and analyzed by software with objective standard.

Reporting for specific materials, systems and methods

We require information from authors about some types of materials, experimental systems and methods used in many studies. Here, indicate whether each material, system or method listed is relevant to your study. If you are not sure if a list item applies to your research, read the appropriate section before selecting a response.

Materials & experimental systems

n/a	Involved in the study
☐	☒ Antibodies
☐	☒ Eukaryotic cell lines
☒	☐ Palaeontology and archaeology
☐	☒ Animals and other organisms
☒	☐ Clinical data
☒	☐ Dual use research of concern

Methods

n/a	Involved in the study
☒	☐ ChIP-seq
☐	☒ Flow cytometry
☒	☐ MRI-based neuroimaging

Antibodies

Antibodies used

APC anti-mouse CD3 Antibody (BioLegend; Catalog number: 100236; Clone name: 17A2; 1:100 dilution)
FITC anti-mouse CD4 Antibody (BioLegend; Catalog number: 100405; Clone name: GK1.5; 1:400 dilution)
PE anti-mouse CD8a Antibody (BioLegend; Catalog number: 100708; Clone name: 53-6.7; 1:200 dilution)
PE anti-mouse CD4 Antibody (BioLegend; Catalog number: 100407; Clone name: GK1.5; 1:200 dilution)
Alexa Fluor® 647 anti-mouse FOXP3 Antibody(BioLegend; Catalog number: 126408; Clone name: MF-14; 1:100 dilution)
FITC anti-mouse CD62L Antibody(BioLegend; Catalog number: 104405; Clone name: MEL-14; 1:400 dilution)
PerCP/Cyanine5.5 anti-mouse/human CD44 Antibody(BioLegend; Catalog number: 103031; Clone name: IM7; 1:200 dilution)
PE anti-mouse CD49b (pan-NK cells) Antibody(BioLegend; Catalog number: 108907; Clone name: DX5; 1:200 dilution)
APC anti-mouse IFN-γ Antibody(BioLegend; Catalog number: 505810; Clone name: XMG1.2; 1:50 dilution)
FITC anti-mouse CD11c Antibody(BioLegend; Catalog number: 117306; Clone name:N418; 1:400 dilution)
PE anti-mouse CD80 Antibody(BioLegend; Catalog number: 104707; Clone name: 16-10A1; 1:100 dilution)
APC anti-mouse CD86 Antibody(BioLegend; Catalog number: 105011; Clone name: GL-1; 1:200 dilution)
PE anti-mouse CD11c Antibody(BioLegend; Catalog number: 117308; Clone name:N418; 1:200 dilution)
FITC anti-mouse CD80 Antibody(BioLegend; Catalog number: 104706; Clone name: 16-10A1; 1:100 dilution)
PerCP/Cyanine5.5 anti-mouse CD86 Antibody(BioLegend; Catalog number: 105028; Clone name: GL-1; 1:50 dilution)
Calreticulin Polyclonal Antibody(Bioss; Catalog: bs-5913R; 1:200 dilution)
Rabbit Anti-HMGB1 antibody (Bioss;Catalog:bs-0664R; 1:200 dilution)
Goat Anti-Rabbit IgG/Alexa Fluor 594 antibody (Bioss;Catalog:bs-0295G-AF594; 1:400 dilution)
Goat Anti-Rabbit IgG/Alexa Fluor 488 antibody (Bioss;Catalog:bs-0295G-AF488; 1:400 dilution)

Validation

All antibodies were well-recognized in the field and have their validation statement on their manufactures' websites: https://www.biolegend.com, https://www.biossusa.com/. Furthermore, these antibodies were validated by data provided in the manuscript.
1.APC anti-mouse CD3 Antibody has been validated to be used for flow cytometric analysis and mentioned species reactivity with mouse. (https://www.biolegend.com/en-us/products/apc-anti-mouse-cd3-antibody-8055);
2.FITC anti-mouse CD4 Antibody has been validated to be used for flow cytometric analysis and mentioned species reactivity with mouse. (https://www.biolegend.com/en-us/products/fitc-anti-mouse-cd4-antibody-248);
3.PE anti-mouse CD8a Antibody has been validated to be used for flow cytometric analysis and mentioned species reactivity with mouse.(https://www.biolegend.com/en-us/products/pe-anti-mouse-cd8a-antibody-155);
4.PE anti-mouse CD4 Antibody has been validated to be used for flow cytometric analysis and mentioned species reactivity with mouse.(https://www.biolegend.com/en-us/products/pe-anti-mouse-cd4-antibody-250);
5.Alexa Fluor® 647 anti-mouse FOXP3 Antibody has been validated to be used for flow cytometric analysis and mentioned species reactivity with mouse.(https://www.biolegend.com/en-us/products/alexa-fluor-647-anti-mouse-foxp3-antibody-4662);
6.FITC anti-mouse CD62L Antibody has been validated to be used for flow cytometric analysis and mentioned species reactivity with mouse.(https://www.biolegend.com/en-us/products/fitc-anti-mouse-cd62l-antibody-384);
7.PerCP/Cyanine5.5 anti-mouse/human CD44 Antibody has been validated to be used for flow cytometric analysis and mentioned species reactivity with mouse.(https://www.biolegend.com/en-us/products/percp-cyanine5-5-anti-mouse-human-cd44-antibody-5605);
8.PE anti-mouse CD49b (pan-NK cells) Antibody has been validated to be used for flow cytometric analysis and mentioned species reactivity with mouse.(https://www.biolegend.com/en-us/products/pe-anti-mouse-cd49b-pan-nk-cells-antibody-234);
9.APC anti-mouse IFN-γ Antibody has been validated to be used for flow cytometric analysis and mentioned species reactivity with mouse.(https://www.biolegend.com/en-us/products/apc-anti-mouse-ifn-gamma-antibody-993);
10.FITC anti-mouse CD11c Antibody has been validated to be used for flow cytometric analysis and mentioned species reactivity with mouse.(https://www.biolegend.com/en-us/products/fitc-anti-mouse-cd11c-antibody-1815);
11.PE anti-mouse CD80 Antibody has been validated to be used for flow cytometric analysis and mentioned species reactivity with mouse.(https://www.biolegend.com/en-us/products/pe-anti-mouse-cd80-antibody-43);
12.APC anti-mouse CD86 Antibody has been validated to be used for flow cytometric analysis and mentioned species reactivity with mouse.(https://www.biolegend.com/en-us/products/apc-anti-mouse-cd86-antibody-2896);
13.PE anti-mouse CD11c Antibody has been validated to be used for flow cytometric analysis and mentioned species reactivity with mouse.(https://www.biolegend.com/en-us/products/pe-anti-mouse-cd11c-antibody-1816);
14.FITC anti-mouse CD80 Antibodyhas been validated to be used for flow cytometric analysis and mentioned species reactivity with mouse.(https://www.biolegend.com/en-us/products/fitc-anti-mouse-cd80-antibody-41);
15.PerCP/Cyanine5.5 anti-mouse CD86 Antibodyhas been validated to be used for flow cytometric analysis and mentioned species reactivity with mouse.(https://www.biolegend.com/en-us/products/percp-cyanine5-5-anti-mouse-cd86-antibody-4276);
16.Calreticulin Polyclonal Antibody has been validated to be used for immunohistochemical staining and mentioned species reactivity with mouse. (http://www.bioss.com.cn/prolook_03.asp?id=AF08169606009769&pro37=1);
17.Rabbit Anti-HMGB1 antibody has been validated to be used for immunohistochemical staining and mentioned species reactivity with mouse.(http://www.bioss.com.cn/prolook_03.asp?id=AF08169606000459&pro37=1).

Eukaryotic cell lines

Policy information about cell lines and Sex and Gender in Research

Cell line source(s)	Mouse malignant melanoma cells (B16F10), mouse colorectal cancer cells (CT26, MC38) and human umbilical vein endothelial cells (HUVECs) were obtained from the Chinese Academy of Sciences (China).
Authentication	The cell lines were not validated because the cell lines were used without modification after purchased and the cell morphology and behavior were consistent with expectations.
Mycoplasma contamination	All cell lines showed negative for mycoplasma contamination.
Commonly misidentified lines (See ICLAC register)	No commonly misidentified cell lines were used in this study.

Animals and other research organisms

Policy information about studies involving animals; ARRIVE guidelines recommended for reporting animal research, and Sex and Gender in Research

Laboratory animals	Female C57BL/6 mice (6-8 weeks) were purchased from SPF Biotechnology Co., Ltd. (Beijing). Female BALB/c mice (6-8 weeks) were purchased from Beijing Vital River Laboratory Animal Technology Co., Ltd. Mice were housed under conditions of a light/dark cycle of 12 h, an ambient temperature of 25 ± 2 °C, and ahumidity of 60 ± 10%.
Wild animals	No wild animals were involved in this study.
Reporting on sex	The sex was not considered in the study design because there was no direct correlation between the selected tumour model and sex.
Field-collected samples	No field-collected samples were involved in this study.
Ethics oversight	All relevant animal experiments were performed in compliance with the animal management rules of the Ministry of Health, People's Republic of China and Animal Experiment Ethics Review Board of Shandong University.

Note that full information on the approval of the study protocol must also be provided in the manuscript.

Flow Cytometry

Plots

Confirm that:

☒ The axis labels state the marker and fluorochrome used (e.g. CD4-FITC).

☒ The axis scales are clearly visible. Include numbers along axes only for bottom left plot of group (a 'group' is an analysis of identical markers).

☒ All plots are contour plots with outliers or pseudocolor plots.

☒ A numerical value for number of cells or percentage (with statistics) is provided.

Methodology

Sample preparation	The sample preparation was described in the Methods.
Instrument	BD Accuri C6 Plus and Beckman Coulter CytoFLEX S were were used for flow cytometry data collection.
Software	BD Accuri C6 Plus and Beckman Coulter CytoFLEX S software were used to collect the data. FlowJo 10 software was used to analyse the data.
Cell population abundance	No sorting was performed by flow cytometry.
Gating strategy	Cells were gated on FSC/SSC in general.

☒ Tick this box to confirm that a figure exemplifying the gating strategy is provided in the Supplementary Information.
